# Synergistic Inhibitory Effect of Quercetin and Cyanidin-3O-Sophoroside on ABCB1

**DOI:** 10.3390/ijms241411341

**Published:** 2023-07-12

**Authors:** Kuljeet Singh, Rajesh B. Patil, Vikas Patel, Judit Remenyik, Tamás Hegedűs, Katalin Goda

**Affiliations:** 1 Department of Biophysics and Cell Biology, Faculty of Medicine, University of Debrecen, 4032 Debrecen, Hungary; 2 Doctoral School of Molecular Cell and Immune Biology, University of Debrecen, 4032 Debrecen, Hungary; 3 Department of Pharmaceutical Chemistry, Sinhgad Technical Education Society’s Sinhgad College of Pharmacy, OffSinhgad Road, Vadgaon (Bk), Pune 411041, Maharashtra, India; 4 Institute of Food Technology, Faculty of Agricultural and Food Sciences and Environmental Management, University of Debrecen, 4032 Debrecen, Hungary; 5 Department of Biophysics and Radiation Biology, Semmelweis University, 1085 Budapest, Hungary; 6 ELKH-SE Biophysical Virology Research Group, Eötvös Loránd Research Network, 1052 Budapest, Hungary

**Keywords:** ABCB1, ATPase activity, transport activity, UIC2 reactivity, substrate–ABCB1 interactions, molecular dynamics (MD) simulations

## Abstract

The human ABCB1 (P-glycoprotein, Pgp) protein is an active exporter expressed in the plasma membrane of cells forming biological barriers. In accordance with its broad substrate spectrum and tissue expression pattern, it affects the pharmacokinetics of numerous chemotherapeutic drugs and it is involved in unwanted drug–drug interactions leading to side effects or toxicities. When expressed in tumor tissues, it contributes to the development of chemotherapy resistance in malignancies. Therefore, the understanding of the molecular details of the ligand–ABCB1 interactions is of crucial importance. In a previous study, we found that quercetin (QUR) hampers both the transport and ATPase activity of ABCB1, while cyandin-3O-sophroside (C3S) stimulates the ATPase activity and causes only a weak inhibition of substrate transport. In the current study, when QUR and C3S were applied together, both a stronger ATPase inhibition and a robust decrease in substrate transport were observed, supporting their synergistic ABCB1 inhibitory effect. Similar to cyclosporine A, a potent ABCB1 inhibitor, co-treatment with QUR and C3S shifted the conformational equilibrium to the “inward-facing” conformer of ABCB1, as it was detected by the conformation-selective UIC2 mAb. To gain deeper insight into the molecular details of ligand–ABCB1 interactions, molecular docking experiments and MD simulations were also carried out. Our in silico studies support that QUR and C3S can bind simultaneously to ABCB1. The most favourable ligand–ABCB1 interaction is obtained when C3S binds to the central substrate binding site and QUR occupies the “access tunnel”. Our results also highlight that the strong ABCB1 inhibitory effect of the combined treatment with QUR and C3S may be exploited in chemotherapy protocols for the treatment of multidrug-resistant tumors or for improving drug delivery through pharmacological barriers.

## 1. Introduction

The human ABCB1 (P-glycoprotein, Pgp) is one of the most studied ABC transporters. It is a plasma membrane resident active transporter with an extremely broad substrate spectrum. It is expressed in many cell types forming pharmacological barriers, such as the intestinal epithelium, blood–brain barrier, blood–testis barrier, hepatocytes and kidney cells, and therefore it is an important determinant of the pharmacokinetics of many drugs [1,2,3]. Since ABCB1 is often expressed in tumor cells and in tumor stem cells, it contributes to the chemotherapy resistance of tumors, referred as multidrug resistance [4,5].

ABCB1 belongs to the group of type I ABC exporters based on its sequence homology and structure [6]. It is a full transporter consisting of two transmembrane domains (TMDs) and two nucleotide-binding domains (NBDs). Structural studies suggest that, similar to other type I ABC transporters, ABCB1 follows the alternating access mechanism, changing conformation between an “inward-facing” state possessing a substrate-binding pocket accessible from the cytosol and an “outward-facing” state presumably involved in substrate release to the extracellular milieu [7,8]. ABCB1 is thought to have arisen from an ancient half-transporter by a gene duplication event followed by randomly occurring point mutations during its evolution, resulting in a pseudo-symmetric protein [9]. Both TMDs contribute to the formation of the substrate-binding cavity [7,10,11].

Numerous research projects have aimed to understand the mechanism of poly-specific ligand recognition by ABCB1. The photolabeling of ABCB1 with substrate analogues such as (^125^I)-iodoarylazidoprazosine (IAAP) or propafenone derivatives suggested that the substrate-binding pocket consists of at least two distinct regions involved in substrate binding [12,13]. Based on studying the kinetics of ABCB1-mediated drug transport, Shapiro et al. supposed the presence of three drug-binding regions that are specific to particular substrates, namely, the H-site (for Hoechst 33342), the R-site (for rhodamine 123) and the P-site (for prazosin and progesterone) [14]. Cysteine mutagenesis experiments combined with thiol-reactive ABCB1 substrates were used to identify amino acid residues involved in substrate binding. These studies proved that the binding sites of several drugs, including cyclosporine A (CsA), tariquidar and valinomycin, spatially overlap with each other and lead to the formulation of the “substrate-induced fit” model, involving the idea that the complex substrate-binding pocket can accommodate structurally different substrate molecules by exposing different amino acid residues on the surface of the pocket, as a result of substrate-specific rotational and lateral movements of the involved TM segments [15,16].

Taken together, these early findings strongly support the existence of multiple substrate-binding sites in the substrate-binding pocket of ABCB1. However, these studies could not determine the exact number of simultaneously bound substrates and could not specify the spatial localization of these binding sites. More recently, X-ray crystallography and cryo-EM studies showed that ligands bind to partially overlapping sites inside of the large central binding cavity formed by the 1st, 4th, 5th and 6th helices of TMD1, and the 7th 10th, 11th and 12th helices of TMD2 [10,11,17]. In addition, the structures of the inhibitor–ABCB1 complexes suggest that inhibitor molecules often bind in pairs, fill up the substrate-binding cavity and form a larger number of interactions with the neighbouring amino acid residues as compared to substrates. These high-affinity ligand–ABCB1 interactions may have long-range effects on the protein conformation that can hinder or even prevent NBD-dimerization and the subsequent steps of the catalytic cycle [10,18].

Active transporters hydrolyze ATP to cover the energy demand of up-hill transport. A common characteristic feature of several ABC transporters including ABCB1 is that they exhibit a weak ATP hydrolytic activity even in the absence of externally added substrates, often referred to as basal ATPase activity. The basal ATP hydrolysis activity of ABCB1 is typically augmented by transported substrates and hampered by inhibitors [2,3].

In a previous study elucidating the effect of dietary polyphenols on ABCB1, we proved the earlier finding that quercetin (QUR) is an ABCB1 inhibitor [19]. QUR decreased both the basal and the substrate-stimulated ATPase activity of ABCB1, and thus increased the intracellular accumulation of calcein and daunorubicin [20]. We also found that cyandin-3O-sophroside (C3S), an anthocyanin compound, increased the basal ATPase activity of ABCB1, while it caused only a weak inhibition of calcein-AM and daunorubicin transport. Interestingly, in our current study, co-treatment with C3S and QUR brought about a strong inhibition of the ABCB1-mediated transport and ATPase activity alike. In addition, co-treatment with QUR and C3S shifted the equilibrium to the “inward-facing” conformer of ABCB1. To gain deeper insight into the molecular details of ligand–ABCB1 interactions, molecular docking experiments and molecular dynamics (MD) simulations were carried out using the zosuquidar (ZQU)-bound cryo-EM structure of ABCB1. The ABCB1 inhibitor ZQU is bound in a pair, with one molecule lodged in the central drug-binding pocket and a second molecule extending into a phenylalanine-rich cavity termed the “access tunnel” [17]. Our in silico studies demonstrate that a similar binding mode, when C3S binds to the central substrate binding site and QUR occupies the “access tunnel”, is likely responsible for their strong and synergistic inhibitory effect.

## 2. Results

### 2.1. Co-Treatment with Quercetin and Cyanidin-3O-Sophoroside Inhibits Human ABCB1-Mediated Transport

**Figure 1:**
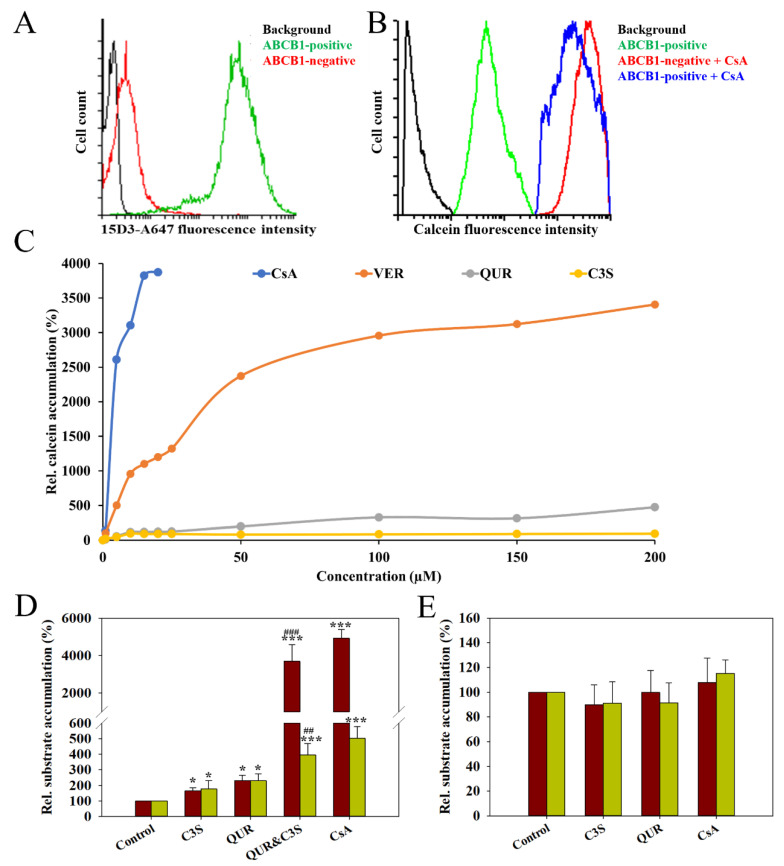
15D3-A647 anti-ABCB1 mAb staining (**A**) and calcein uptake (**B**) of NIH 3T3 and NIH 3T3 MDR1 cells. Concentration-dependent effects of quercetin (QUR), cyanidin-3O-sophoroside (C3S), verapamil (VER) and cyclosporine A (CsA) on the calcein accumulation in NIH 3T3 MDR1 cells (**C**). The effects of C3S and QUR on the calcein (red bars) and daunorubicin (green bars) accumulation in NIH 3T3 MDR1 (**D**) and NIH 3T3 (**E**) cells. Cells were pre-treated with CsA (10 µM) QUR (100 µM), C3S (100 µM) or their combination for 10 min at 37 °C and then further incubated with ABCB1 substrates (0.5 µM calcein-AM or 3.8 µM daunorubicin) for 20 min and 30 min, respectively. The intracellular accumulation of calcein and daunorubicin was measured in a flow cytometer. Relative substrate accumulation was calculated by normalizing the mean calcein or daunorubicin fluorescence intensities of the polyphenol/CsA-treated samples to the untreated control samples. Bars represent the means (±SD) of at least three independent measurements. Significant differences compared to untreated samples are shown by ***—*p* < 0.001 and *—*p* < 0.05, while significant differences between QUR- and QUR and C3S-treated samples are indicated by ###—*p* < 0.001 and ##—*p* < 0.01.

To study the effects of quercetin (QUR), cyanidin-3O-sophoroside (C3S) and their combination on the transport activity of human ABCB1, calcein and daunorubicin accumulation experiments were carried out using the NIH 3T3 (ABCB1-negative) and NIH 3T3 MDR1 (ABCB1-positive) cell lines. NIH 3T3 MDR1 cells express human ABCB1 at very high levels in their plasma membrane, as demonstrated by 15D3-A647 mAb staining, while NIH 3T3 cells show only a negligible 15D3-reactivity (Figure 1A). Accordingly, the ABCB1-positive cells exhibited very weak calcein accumulation, which was elevated almost to the level of ABCB1-negative cells by 10 µM cyclosporine A (CsA) (Figure 1B) in agreement with the robust ABCB1 inhibitory effect of CsA demonstrated previously [21,22]. Similar to CsA, verapamil (VER) also strongly increased the calcein accumulation in ABCB1-positive cells, albeit with different concentration dependence (Figure 1C). Importantly, compared to CsA and VER, QUR and C3S had only a weak effect on the transport activity of ABCB1 (Figure 1C). However, interestingly, the combination of QUR and C3S both added at 100 µM concentration brought about a strong transport inhibitory effect in the calcein and daunorubicin accumulation experiments. The combined effect of QUR and C3S was greater than the sum of their individual effects in both assays (Figure 1D). The observed inhibitory effects are ABCB1-specific, since the above compounds did not have any significant effect on calcein and daunorubicin accumulation by the ABCB1-negative NIH 3T3 cells (Figure 1E).

### 2.2. Quercetin and Cyanidin-3O-Sophoroside Synergistically Lower the ATPase Activity of ABCB1

**Figure 2:**
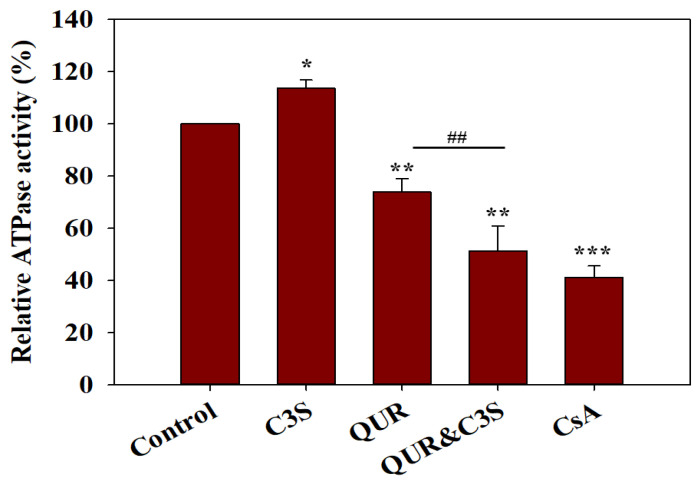
Synergistic inhibitory effects of quercetin (QUR) and cyanidin-3O-sophoroside (C3S) on the ATPase activity of ABCB1. QUR and C3S were applied at 100 µM, while the known ATPase inhibitor cyclosporine A (CsA) was used at a 10 µM concentration. Relative ABCB1-specific ATPase activities were calculated relative to the untreated samples. Bars represent the means (±SD) of three independent measurements, each performed in triplicates. Significant differences compared to untreated control are shown by ***—*p* < 0.001, **—*p* < 0.01 and *—*p* < 0.05, while significant differences between QUR and the QUR and C3S combination are shown by ##—*p* < 0.01.

The transported substrates generally increase the basal ATPase activity of ABC transporters, while transport inhibitors affect the ATPase activity depending on their mechanism of action [23]. When applied alone, QUR decreased the ATPase activity of ABCB1, while C3S weakly stimulated it. Interestingly, a strong inhibitory effect was observed when these compounds were added simultaneously (Figure 2).

### 2.3. Synergistic Effects of Quercetin and Cyanidin-3O-Sophoroside on the Conformational Transitions Detected by UIC2

**Figure 3:**
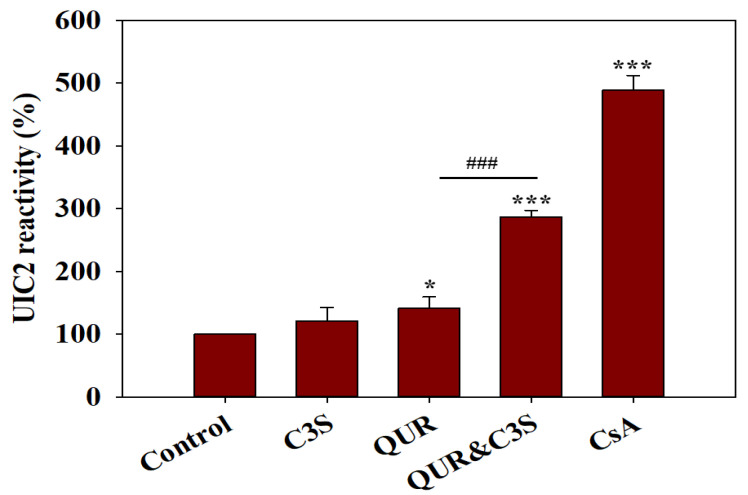
Effects of quercetin (QUR) and cyanidin-3O-sophoroside (C3S) on the UIC2-reactivity of ABCB1 expressed in NIH 3T3 cells. Cells were pre-incubated in the presence or absence of cyclosporine A (CsA, 10 µM), QUR, C3S or their combination for 10 min at 37 °C and then further incubated with 10 µg/mL UIC2-A647 for another 30 min. The UIC2-A647 staining of polyphenol/CsA-treated cells was normalized to that of the untreated cells. Bars represent the means (±SD) of at least three independent measurements. Significant differences compared to untreated samples are shown by ***—*p* < 0.001 and *—*p* < 0.05, while significant differences between QUR and QUR and C3S treatments are shown by ###—*p* < 0.001.

Previous studies have suggested that efficient transport inhibitors stabilize ABCB1 molecules in a UIC2-reactive conformational state [24]. Therefore, to further investigate the interaction of ABCB1 with QUR and C3S, UIC2 reactivity assays were carried out. When added individually, the two polyphenols hardly affected the UIC2 reactivity of NIH 3T3 MDR1 cells. However, an almost three-fold increase in the UIC2 reactivity was detected when the two compounds were applied simultaneously, in accordance with their strong ABCB1-inhibitory effect (Figure 3).

### 2.4. Molecular Docking Experiments

**Figure 4:**
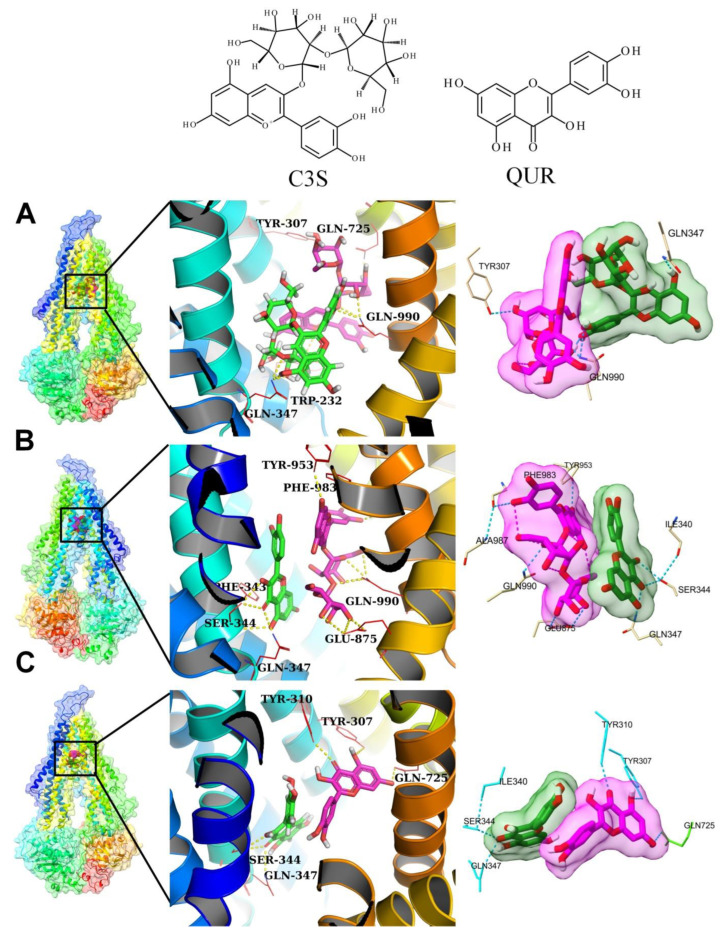
The best docked poses of C3S and QUR in the substrate-binding pocket of ABCB1. (**A**) ABCB1 complex with C3S at both binding sites (2×C3S–ABCB1 complex); (**B**) ABCB1 complex with C3S at site 1 and QUR at site 2 (C3S-QUR–ABCB1 complex) and (**C**) ABCB1 complex with QUR at both sites (2×QUR–ABCB1 complex). Ligands at site 1 are shown in magenta (stick and surface representations), ligands at site 2 are depicted in green, while the TM helices are shown in a rainbow colour scheme.

The observed synergistic effect of the co-treatment with QUR and C3S supports the notion that they may bind simultaneously to the large substrate-binding pocket of ABCB1. Based on the cryo-EM structure of human ABCB1 in complex with two molecules of the inhibitor ZQU (PDB ID: 7A6F, resolution: 3.50 Å), two ligand-binding sites were identified: the central binding site (site 1) and the “access tunnel” (site 2) [17]. To understand the molecular details of the interaction between ABCB1 and the ligands QUR and C3S, in silico molecular docking studies were carried out using AutoDock Vina [25]. The docking protocol was validated as shown in Figures S1 and S2 and in the corresponding Supplementary Text. To find the best poses of ligands with the lowest binding free energy, we individually docked C3S and QUR to site 1 and site 2, as is described in Figure S3 and in the corresponding Supplementary Text. The best poses of the different ligand pairs in the substrate-binding cavity, from here on referred as ligand–ABCB1 complexes, are shown in Figure 4.

### 2.5. Molecular Dynamics Simulations

Compared to in silico docking, the MD simulations provide a more accurate estimation for the ligand binding affinities and the overall stability of the studied system, since the flexibility of residue side chains and the protein backbone can also be taken into account [26]. Therefore, we performed MD simulations to gain further insights into the dynamics of TM segments of ABCB1 in the presence of ligand pairs.

#### 2.5.1. Analysis of Hydrogen Bonding between Ligands and Amino Acid Residues of the Substrate-Binding Pocket

Previous studies suggest that the formation of hydrogen bonds may have crucial importance in the potent inhibitory effects of certain ABCB1 ligands, such as CsA, ZQU, elacridar and tariquidar [27,28]; therefore, we analyzed the hydrogen bond interactions in the three ligand–ABCB1 complexes.

##### 2×C3S–ABCB1 Complex

In ABCB1 with two docked molecules of C3S (2×C3S–ABCB1 complex), the C3S molecule at site 1 showed hydrogen bond interactions with residues Tyr307, Gln990, and Gln725, which are contributing to the central binding site (site 1), while C3S at site 2 formed a hydrogen bond interaction with Gln347 and Trp232 (Figure 4A and Figure S4A). The equilibrated trajectory from the run with the lowest average RMSD (run 2) showed that the hydrogen bond interactions with Tyr307, Gln725 and Gln990, present in the docked pose at site 1, and with Trp232 and Gln347 at site 2, were broken, and new hydrogen bond interactions were formed with Ser344 and Glu875 at site 1 and Gln990, Tyr307 and Tyr310 at site 2 (Figure S4B). However, the trajectory at 300 ns showed the re-establishment of hydrogen bonds with Tyr307 and Gln725 at site 1, and a new hydrogen bond was formed with Tyr953 at site 2 (Figure S4C). The analysis of conformations extracted from the trajectories at 325, 350, 375 and 400 ns corroborated the docking results and highlighted that Gln725 and Tyr307 at site 1 and residues Tyr953 at site 2 are the key residues in hydrogen bond formation (Figures S4C and S5 and Supplementary Text).

However, these results are specific to the conformations extracted at different time intervals of MD simulations. Therefore, we calculated hydrogen bond occupancy to be able to draw more robust conclusions regarding crucial hydrogen bonds. The network of hydrogen bond pairs with a threshold of 15% occupancy was calculated in the concatenated trajectory between the 300 ns and 400 ns simulation periods, and is shown in Table 1. Residue Gln725 at site 1 formed a stable hydrogen bond with C3S and exhibited the highest occupancy as a hydrogen bond acceptor. Residues Gln838, Ser228, Tyr307 and Tyr310 also formed hydrogen bonds with C3S at site 1 throughout the whole simulation period. At site 2, Tyr953 formed a very stable hydrogen bond with C3S throughout the entire simulation, acting predominantly as a hydrogen bond donor. In addition, residues Gln725, Glu875 and Tyr310 also formed hydrogen bonds with various donor or acceptor atoms of C3S at site 2.

Furthermore, the number of hydrogen bonds formed during the entire MD simulation is also important in characterizing the overall binding interactions between ligands and binding site residues (Figure S6A). The average number of hydrogen bonds at the respective sites in the case of the different ligand pairs is listed in Table S1. C3S formed an average of five and three hydrogen bonds at site 1 and site 2, respectively. When both ligands are considered together, an average of six hydrogen bonds were formed.

The intermolecular non-bonded interactions between the ligands are also important in holding the ligand pair together at the respective binding sites. In the docked poses of the C3S pair, two intermolecular hydrogen bonds were observed (right panel of Figure S4A). Similarly, such intermolecular hydrogen bonds were observed in the equilibrated trajectory, as well as in the trajectories in the 300, 325, 375 and 400 ns simulation period.

##### C3S-QUR–ABCB1 Complex

The docked poses of C3S at site 1 exhibited hydrogen bond interactions with Tyr953, Phe983, Ala987, Gln990 and Glu875, while QUR at site 2 formed hydrogen bonds with Gln347, Ser344 and Ile340 (Figure 4B and Figure S7A). After the six-step equilibration, run 1 showed that hydrogen bonds were formed between C3S at site 1 and residues Gln725, Gln990, Glu875 and Gln347, while no hydrogen bonds were created by QUR at site 2 (Figure S7B). The concatenated trajectory after the 300 ns simulation period showed that C3S formed hydrogen bonds with Gln990, Glu875 and Tyr953, while QUR with Tyr310 and Gln347 (Figure S7C). A detailed analysis of the frames extracted at 325, 350, 375 and 400 ns is shown in Figure S8 and described in the corresponding Supplementary Text.

Hydrogen bond occupancy was also studied to describe the incidence of hydrogen bonds during the entire production phase of MD simulations (Table 2). At site 1, C3S formed a very stable hydrogen bond of the highest occupancy with residue Phe983. Residues Gln990 and Trp232 established hydrogen bonds with different donor or acceptor atoms of C3S. At site 2, QUR created the most stable hydrogen bonds with Tyr310 residues with over 50% occupancy. Taken together, C3S was observed to be involved in forming an average of four hydrogen bonds, while QUR formed around one hydrogen bond. When both ligands were considered together, an average of six hydrogen bonds were formed during the entire length of our MD simulations (Table S1 and Figure S6B). The trajectories extracted at 325, 375 and 400 ns also showed intermolecular hydrogen bonds between C3S and QUR (right panel of Figure S8).

##### 2×QUR–ABCB1 Complex

The docked QUR at site 1 formed hydrogen bond interactions with Tyr310, Tyr307 and Gln725, while QUR at site 2 established hydrogen bonds with residues Ser344 and Gln347 (Figure 4C and Figure S9A). After energy minimization and six steps of equilibration, run 1 showed that QUR at site 1 formed hydrogen bond interactions with Tyr310, Tyr307 and Trp232, suggesting that the hydrogen bonds with residues Tyr310 and Tyr307 are probably stable. On the other hand, QUR at site 2 did not form any hydrogen bonds (Figure S9B). The frame extracted at 300 ns showed a hydrogen bond with Gln725 for QUR at site 1, while QUR at site 2 formed a hydrogen bond with Tyr310 (Figure S9C). Interestingly, all the other trajectories at 325, 350, 375 and 400 showed these hydrogen bonds intact and stable (Figure S10). Furthermore, the hydrogen bond occupancy results (Table 3) suggest that at site 1, the residue Gln725 formed a hydrogen bond with the highest occupancy. At site 2, the residue Tyr310 formed a hydrogen bond with the highest occupancy. In addition, the hydrogen bond with Ser228 at site 2 had an occupancy above 90%, suggesting a stable hydrogen bond, although it was not seen in the aforementioned isolated trajectories.

The system with QUR at both of the sites had the lowest number of hydrogen bonds compared to the other two ligand–ABCB1 complexes. QUR at site 1 and 2 formed averages of only one and two hydrogen bonds, respectively. When both QUR molecules were considered together, an average of three hydrogen bonds were formed, which is significantly lower compared to the two other systems (Table 1, Table 2 and Table S1 and Figure S6C). Interestingly, opposed to the other two ligand–ABCB1 complexes, in the 2×QUR–ABCB1 complex, we did not identify intermolecular hydrogen bond interactions between the ligand molecules (right panel of Figure S10).

##### The Same Ligand Exhibits Different Hydrogen Bonding Patterns in Hetero-Liganded and Homo-Liganded Complexes

**Figure 5:**
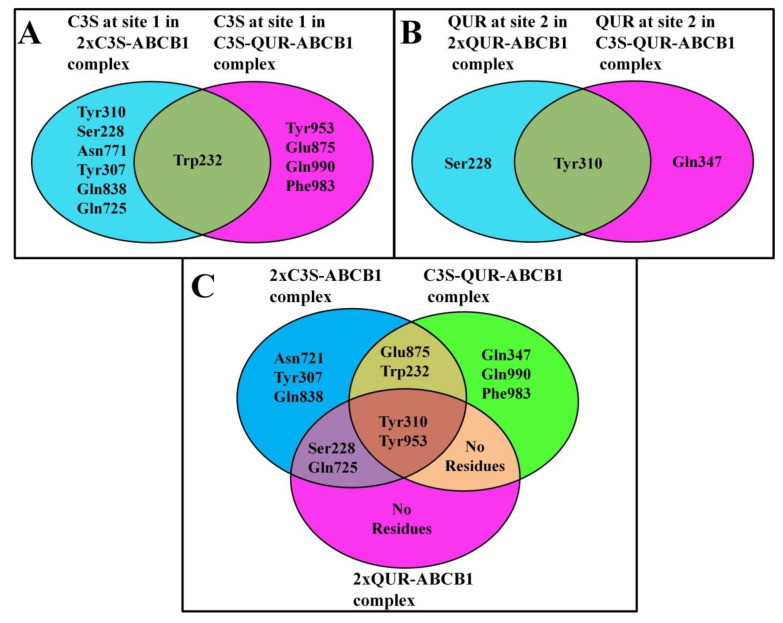
Venn diagrams showing hydrogen bonding partners for C3S and QUR. (**A**) Amino acids interacting with C3S at site 1 in 2×C3S-QUR–ABCB1 and C3S-QUR–ABCB1 complexes. (**B**) Amino acids interacting with QUR at site 2 in 2×QUR–ABCB1 and C3S-QUR–ABCB1 complexes and (**C**) amino acids interacting with the ligands in the 2×C3S–ABCB1, C3S-QUR–ABCB1 and 2×QUR–ABCB1 complexes.

To further understand the interactions of the different ligand combinations with the drug-binding pocket, we compared the hydrogen bonding interaction partners of the ligands in the different complexes using Venn diagrams (Figure 5). Interestingly, the hydrogen bonding interaction partners of C3S at site 1 in the homo-liganded (2×C3S–ABCB1) and hetero-liganded (C3S-QUR–ABCB1) complexes only partially overlap (Figure 5A). The only common hydrogen bonding interaction partner of C3S at site 1 in the 2×C3S–ABCB1 and C3S-QUR–ABCB1 complexes was Trp232. However, C3S additionally interacted with Tyr310, Ser228, Asn771, Tyr307, Gln838, and Gln725 in the 2×C3S–ABCB1 complex. In contrast, this molecule formed hydrogen bonds with Tyr953, Glu875, Gln990 and Phe983 in the C3S-QUR–ABCB1 complex (Figure 5A). A similar phenomenon can be observed regarding the hydrogen bonding interactions of QUR at site 2 in the C3S-QUR–ABCB1 and 2×QUR–ABCB1 complexes, where the residue Tyr310 was the only common hydrogen bonding partner (Figure 5B). The QUR molecule at site 2 interacted with Ser228 in the 2×QUR–ABCB1 complex, while it formed a hydrogen bond with Gln347 in the C3S-QUR–ABCB1 complex (Figure 5B). When the two binding sites were considered together, only two amino acids, namely, Tyr310 and Tyr953, were identified that could form hydrogen bonds with the ligands in every studied ligand–ABCB1 complex (Figure 5C).

#### 2.5.2. Analysis of the Conformation Changes in the NBDs and TMDs of Ligand–ABCB1 Complexes

To investigate the stability of the entire system both the RMSD of protein C-α atoms and the RMSD of ligand atoms were studied in the three ligand-ABCB1 complexes (Figure S11 and Table S1). RMSD values of ABCB1 suggest that the protein structure is the most stable in the 2×C3S-ABCB1 complex (Figure S11A, Table S1), while the conformation of the ligands is the most stable in the C3S-QUR-ABCB1 complex (Figure S11B–D). The atomic fluctuations were more pronounced in the NBDs compared to TMDs (Figures S12A, S13A and S14A), in accordance with previous studies [29,30]. However, the NBD1 dynamics decreased in the presence of QUR both in the 2×QUR–ABCB1 and in the C3S-QUR–ABCB1 complex (Figures S12B, S13B and S14B), suggesting that the stabilization of the NBDs may have contributed to the ATPase inhibitory effect of QUR.

**Figure 6:**
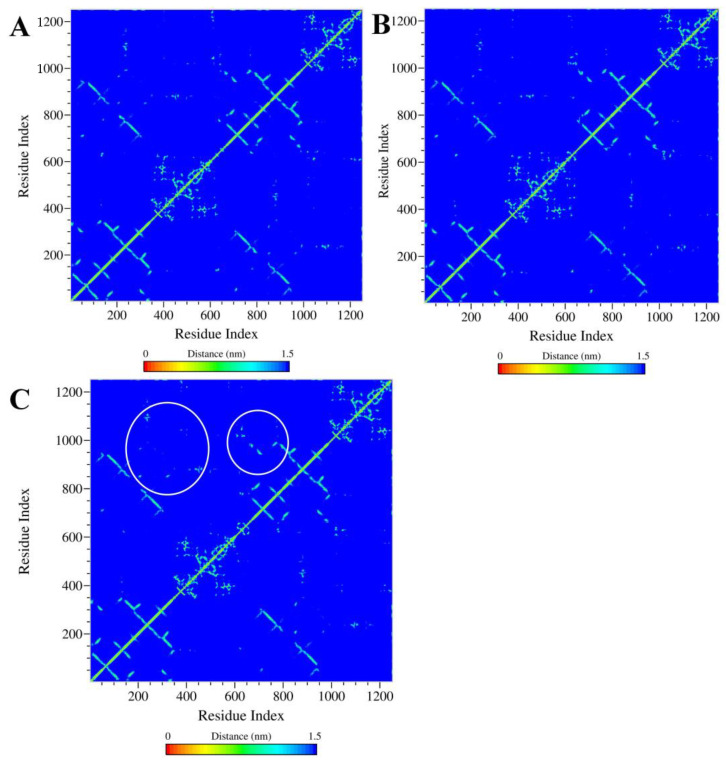
Contact maps for the 2×C3S–ABCB1 (**A**), C3S-QUR–ABCB1 (**B**) and 2×QUR–ABCB1 complexes (**C**).

Contact map analysis provides a two-dimensional representation of the average pair-wise distances between amino acid residues over the MD simulation (Figure 6). The concatenated trajectory of the triplicate-run MD simulations between the 300 ns and 400 ns simulation periods was used for contact map analysis. The analysis shows that residues 200–300 and residues 900–1000, both involved in the formation of the ligand-binding pocket, showed many close contacts with each other, and several residues remained within the distance below 10 Å in the 2×QUR–ABCB1 complex (Figure 6C, white circles). On the other hand, in the C3S-QUR–ABCB1 and 2×C3S–ABCB1 complexes, fewer residue–residue contacts were observed, and the above-described binding site residues remained above the cut-off distance of 15 Å. These data suggest that the total volume of the ligand-binding pocket in 2×QUR–ABCB1 complex was smaller compared to the other two complexes.

**Figure 7:**
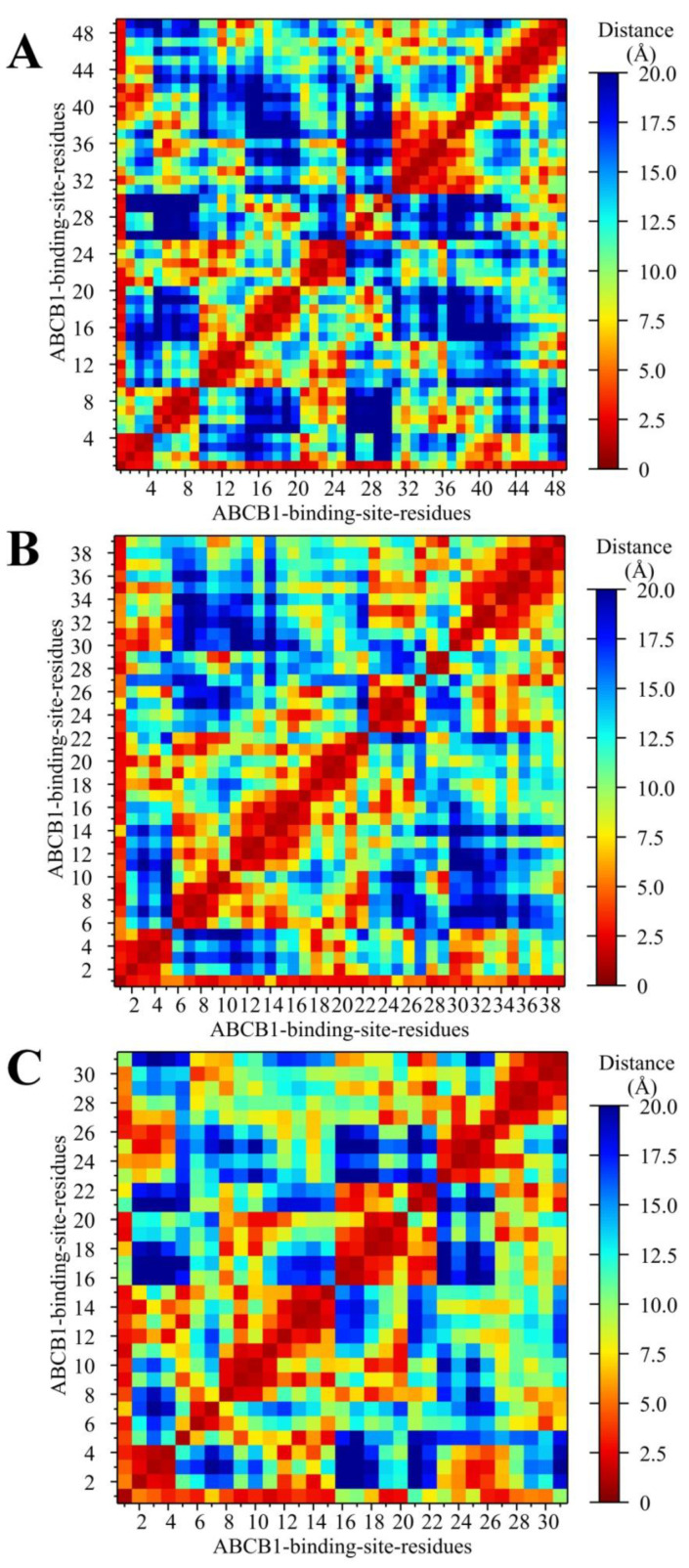
Distance maps for binding site residues within 5 Å from the ligands in the 2×C3S–ABCB1 (**A**), C3S-QUR–ABCB1 (**B**) and 2×QUR–ABCB1 (**C**) complexes. Ligands are considered as residue 1 in distance matrix plots.

The minimum distance between ligands and binding site residues was also analyzed to understand the conformational changes in the binding pocket. A distance matrix for the residues and ligands at both binding sites was generated. From the initial trajectory (300 ns), the residues within 5 Å from the ligands were selected. In the 2×C3S–ABCB1 complex, 48 residues were within 5 Å from the ligands, while 38 and 30 residues were within 5 Å in the C3S-QUR–ABCB1 and 2×QUR–ABCB1 complexes, respectively (Figure 7). It was found that the fewest residues moved beyond 5 Å from the ligand pair in the C3S-QUR–ABCB1 complex compared to the other two complexes during the rest of the simulation period (see distance matrix for residue 1 in Figure 7B), signifying the stability of the ligand-binding pocket interactions in the C3S-QUR–ABCB1 complex.

We also carried out solvent-accessible surface area (SASA) measurements to estimate the area and volume of the buried binding sites in the different ligand–ABCB1 complexes (Figure S15). The SASA values were 9400 Å^2^, 8405 Å^2^ and 7700Å^2^, while the volumes were 35,000, 29,500 and 27,500 Å^3^ for the 2×C3S–ABCB1, C3S-QUR–ABCB1 and 2×QUR–ABCB1 complexes, respectively. These results suggest that the volume of the binding pocket can be accommodated to the size of the bound ligands.

#### 2.5.3. Binding Free Energy of the Different Ligand–ABCB1 Complexes

We applied MM-PBSA calculations to compare the binding affinities of the different ligand pairs to ABCB1. Although the entropic term is neglected in MM-PBSA calculations and therefore these calculations do not provide the real binding free energy values, the obtained free energy values are appropriate for comparing the stability of the different ligand–ABCB1 complexes [30]. The 300–400 ns trajectories extracted at each 250 ps were used to calculate binding free energy (ΔG_bind_) in MM-PBSA calculations (Figures S16–S18 and Table 4). It was observed in these calculations that the van der Waals energies and polar solvation energies of the individual ligands are the most influential in determining the final ΔG_bind_.

**Figure 8:**
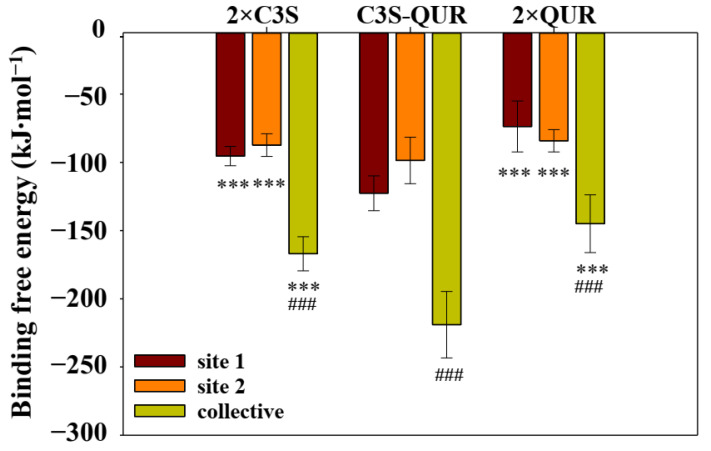
Binding free energy of ligand–ABCB1 complexes. Binding free energy values of ligands were calculated in site 1, in site 2, and when both binding sites were considered together (i.e., collective) on trajectories extracted from the 300 ns to 400 ns MD time intervals with 250 ps steps. Bars represent mean values (±SD, n = 401). Significant differences compared to the C3S-QUR–ABCB1 complex (***: *p* < 0.001). Significant difference of the collective binding free energy compared to site 1 and site 2 (###: *p* < 0.001).

The MM-PBSA results for the 2×C3S–ABCB1 complex show that C3S at site 1 (ΔG_bind_ = −95.41 kJ/mol) and C3S at site 2 (ΔG_bind_ = −87.37 kJ/mol) possess quite similar binding free energies (Table 4 and Figure 8). However, when both ligands were considered together in the MM-PBSA calculation, the corresponding collective binding free energy (ΔG_bind_ = −166.99 kJ/mol) was found to be much lower compared to the individual ligands.

When the systems of C3S at site 1 and QUR at site 2 were subjected to MM-PBSA calculations, the ΔG_bind_ for C3S at site 1 was −122.66 kJ/mol, which is lower compared to C3S at site 1 in the 2×C3S–ABCB1 complex (Figure 8). Similarly, the ΔG_bind_ of QUR at site 2 was −98.60 kJ/mol, which is lower than the ΔG_bind_ of C3S at this site. Most importantly, the collective ΔG_bind_ of the C3S–QUR pair was found to be −218.96 kJ/mol, which is the lowest amongst all the ligand pairs (Figure 8). This result clearly suggests that C3S in combination with QUR bound at site 1 and site 2, respectively, increases the overall binding affinities of these ligands.

When the system with QUR at both sites was subjected to MM-PBSA calculations, the QUR at site 1 and 2 was found to have ΔG_bind_ values of −73.70 and −84.17 kJ/mol, respectively. The collective ΔG_bind_ of this pair of ligands is −144.93 kJ/mol, which is less favorable compared to the C3S–QUR combination.

## 3. Discussion

In the current study we have observed a synergistic inhibitory effect of the co-treatment with C3S and QUR on the transport and ATPase activity of human ABCB1. To study the interaction of C3S and QUR with the substrate binding pocket of ABCB1, we carried out in silico ligand docking and MD simulation experiments. We also analyzed the hydrogen bonds formed between ligands, as well as ligands and binding site residues. However, other weaker non-bonded interactions, such as hydrophobic interactions and π–π stacking, could not be studied, since current MD force fields are unable to capture these subtle electronic behaviours correctly.

Our in silico docking and MD simulation experiments suggest that C3S and QUR may bind simultaneously to the large substrate-binding pocket of ABCB1. The most favorable binding poses were obtained when the bulkier C3S molecule bound to the central substrate binding site and the smaller QUR molecule occupied the “access tunnel”. Moreover, the simultaneous binding of two C3S or two QUR molecules to the complex drug-binding pocket of ABCB1 is energetically less favorable compared to the C3S–QUR combination (Table 4 and Figure 4).

The possibility that more than one ligand molecule can bind simultaneously to the substrate-binding cavity was suggested before the cryo-EM technique revolutionized the field, and ligand-bound ABCB1 structures appeared [10,11]. Many substrates or competitive inhibitors possess bell-shaped ATPase activity vs. ligand concentration curves that are in a perfect agreement with a two-site binding model [31]. Accordingly, previous research suggested that elacridar and tariquidar can be transported by ABCB1 when they are present at very low concentrations [32] and only a single molecule occupies the binding pocket. At higher (micromolar) concentrations, the equilibrium is presumably shifted to the simultaneous binding of two molecules. In this case, the second molecule binds to the “access tunnel” surrounded by TM5, TM7, TM8, TM9 and TM12 helices, and probably behaves as a non-competitive inhibitor [11]. In accordance with this hypothesis, certain drugs (e.g., azidothymidine, paliperidone, abacavir) are efficiently transported by ABCB1 in their monomeric form, while they become high-affinity inhibitors when two identical molecules are chemically cross-linked [33,34]. In another study, flavonoid homo- and heterodimers were synthesized and were found to be more potent ABCB1-inhibitors compared to the original monomeric compounds [35].

In the MD simulations, we observed about a 1.5-fold difference between the collective binding free energies of the homo-liganded ABCB1 complexes (ΔG_bind_ = −144.930 kJ∙mol^−1^ for the 2×QUR–ABCB1complex and ΔG_bind_ = −166.995 kJ∙mol^−1^ for the 2×C3S–ABCB1 complex) and the hetero-liganded ABCB1 complex (ΔG_bind_ = −218.960 kJ∙mol^−1^ for C3S-QUR–ABCB1). Although the formation of the C3S-QUR–ABCB1 complex seems to be energetically more favorable and probably more stable, the binding energy differences alone cannot explain the stronger inhibitory effect of the C3S–QUR combination. However, we also observed that co-treatment with C3S and QUR strongly increases the UIC2 reactivity, suggesting that this ligand combination hinders the function-dependent conformational changes of ABCB1 (Figure 3). This finding is in accordance with the previous observations that the most potent ABCB1 inhibitors stabilize the UIC2-reactive conformation of the transporter [24,36]. It is plausible that the larger number and higher occupancy of the hydrogen bonds formed in the case of the C3S-QUR–ABCB1 complex compared to the 2×QUR–ABCB1 complex may contribute to the conformational stabilization of the transporter (Table 2 and Table 3). Although two C3S molecules could form on average six hydrogen bonds with amino acids lining the drug-binding pocket, similar to the C3S–QUR combination (Table 1), the 2×C3S–ABCB1 complex is probably less stable, probably due to the steric constraints imposed by the bulkier C3S molecules. In addition, it is also possible that C3S could not saturate both binding sites in our live cell experiments (Figure 1 and Figure 3) and ATPase assays (Figure 2). As C3S is a di-glucoside derivative, it may have lower membrane permeability compared to QUR, and thus it may reach lower concentrations in the cytosol and in the plasma membrane from where ligands can access the substrate-binding sites of ABCB1.

A recent study using hydrogen deuterium exchange mass spectrometry (HDX-MS) demonstrated that ligands having different effects on the ATPase activity of ABCB1 differentially modulate the dynamics of the NBDs and intracellular helixes that are far from their binding sites [23]. Similarly, we have observed reduced RMSF in the conserved motifs of NBD1 (Figures S12–S14) in ligand–ABCB1 complexes showing decreased ATPase activity (Figure 2). These observations raise the question of whether the binding of transported and inhibitory ligands to the substrate-binding cavity can induce different conformational changes in the NBDs. However, further clarification of these details requires longer MD simulations in the presence of ATP/Mg^2+^ and different ATPase stimulatory and inhibitory ligands.

When analyzing the hydrogen-bonding interactions between the ligands and amino acid residues of the ligand-binding sites, we observed that Tyr310 and Tyr953 were hydrogen bond donors in all of the three studied complexes (Table 1, Table 2 and Table 3 and Figure 5C). The crucial importance of these residues in ligand binding is also supported by the cryo-EM structure of the zosuquidar–ABCB1 complex [10]. Several in silico docking and MD simulation studies demonstrated that these residues may also form hydrogen bonds with tariquidar and elacridar [28], benzophenone sulphonamide derivatives [37] and other known ABCB1 ligands including CsA, carvedilol or doxorubicin [30]. The hydrogen bonding interactions in the three polyphenol–ABCB1 complexes suggest that the pattern of hydrogen bonds of a particular ligand at one site is affected by the identity of the ligand occupying the other binding site (Table 1, Table 2 and Table 3 and Figure 5A,B). This finding supports the idea that the binding of one ligand may induce specific rotational and lateral movements of the involved TM segments, thus also affecting the binding mode of the other ligand in accordance with the “substrate-induced fit model” [15]. In addition, contact map analysis (Figure 6 and Figure 7) as well as SASA measurements (Figure S15) confirmed that the volume of the substrate-binding pocket can be accommodated to the size of the ligands, further supporting the inherent flexibility of the TM segments forming the substrate-binding pocket [38].

## 4. Materials and Methods

### 4.1. Chemicals

All chemicals, cell culture media and supplements used for the study were purchased from Sigma-Aldrich (Budapest, Hungary). Fluorescent dyes including calcein acetoxy methylester (calcein-AM) and Alexa 647 succinimidyl ester (A647) were obtained from Life Technologies Inc. (Carlsbad, CA, USA). 15D3 and UIC2, the ABCB1-specific monoclonal antibodies (mAbs), were purified from hybridoma supernatants by affinity chromatography [39]. The antibody-producing hybridoma cell lines were obtained from the American Type Culture Collections (Manassas, VA, USA). Abs were conjugated with A647 and then separated from the unconjugated dye by gel filtration using a Sephadex G-50 column. The dye-to-protein labeling ratio was around 3 for every antibody preparation. Verapamil was dissolved in water, while calcein-AM, cyclosporine A (CsA), quercetin (QUR) and cynidin-3-O-sophroside (C3S) were dissolved in DMSO according to the manufacturer’s instructions. For all experiments, the final DMSO concentration of samples was less than 1% (*v*/*v*).

### 4.2. Cell Culturing

The NIH 3T3 mouse fibroblast cell line and its human ABCB1-overexpressing counterpart (NIH 3T3 MDR1) [40] were kindly provided by Michael Gottesman (National Institutes of Health, Bethesda, MD, USA). Cells were maintained in Dulbecco’s modified Eagle’s medium (DMEM) supplemented with 0.1 mg/mL penicillin-streptomycin cocktail, 10% heat-inactivated fetal calf serum and 2 mM L-glutamine. To maintain their multidrug-resistant phenotype, NIH 3T3 MDR1 cells were cultured in the presence of 670 nM doxorubicin and transferred to a doxorubicin-free medium only 2–3 days prior to the experiments. The ABCB1 expression level of cells was regularly checked by staining with 30 µg/mL 15D3-A647 mAb for 30 min at 37 °C. Antibody staining was measured by flow cytometry.

### 4.3. Transport Activity Measurements

For studying the effect of QUR and C3S on the transport activity of ABCB1, we used daunorubicin and calcein accumulation assays [41,42]. Cells harvested at about 80% confluency were washed three times in PBS containing 7 mM glucose (gl-PBS). Samples containing 0.5 × 10^6^ cells/mL were pre-treated with different concentrations of QUR, C3S, QUR + C3S or the known competitive ABCB1 inhibitors CsA or verapamil for 10 min at 37 °C and then further incubated with 3.8 µM daunorubicin for 30 min or 0.5 µM calcein-AM for 20 min. Following three washing steps with ice-cold gl-PBS containing 0.5% FBS, the samples were kept on ice until flow cytometry measurement.

### 4.4. Crude Membrane Preparation for ATPase Activity Measurements

The cells were harvested by scraping them into phosphate-buffered saline (PBS, pH 7.4), and washed twice. Membrane preparation was carried out according to Sarkadi’s method [43], with minor modifications. All procedures were carried out at 4 °C. Cell homogenization was performed using a glass–Teflon tissue homogenizer in TMEP solution (50 mM Tris-HCl (pH 7.0), 50 mM mannitol, 2 mM EGTA, 0.5 mM phenylmethylsulphonyl fluoride (PMSF) and protease inhibitor cocktail (PIC, Sigma-Aldrich, Budapest, Hungary)). Nuclear debris was removed by centrifugation at 500× *g* for 10 min, and the resulting supernatants were centrifuged for 60 min at 28,000× *g*. Finally, the pellet containing cellular membranes was re-suspended in TMEP solution and was stored at −70 °C until use.

### 4.5. ATPase Activity Measurements

The ABCB1-specific ATPase activity of the membrane samples was determined by measuring the amount of inorganic phosphate (Pi) released upon ATP hydrolysis in the presence of inhibitors of other highly expressed ATPases (NaN_3_ for the F0F1 ATPases, ouabain for Na^+^/K^+^-ATPase and EGTA for the Ca^2+^-ATPases) [43]. Membrane samples (5 µg membrane protein/sample) were pre-incubated with the tested compounds (e.g., QUR, C3S) with or without 40 µM verapamil in 60 µL ATPase assay premix (50 mM MOPS, 65 mM KCl, 6.5 mM NaN3, 2.6 mM DTT, 1.28 mM ouabain, 0.65 mM EGTA, pH 7.0) in the presence or absence of 100 µM Na_3_VO_4_ (vanadate) for 5 min at 37 °C. Subsequently the ATPase reaction was initiated by the addition of 3.2 mM ATP/Mg^2+^. After 25 min incubation at 37 °C, the ATPase reaction was stopped by 40 µL 5% SDS, then the samples were incubated with 105 µL color reagent at room temperature for 30 min [43,44]. To determine the amount of released Pi in the samples, absorbance was measured at 700 nm using a BioTek Synergy HT plate reader (BioTek Instruments, Winooski, VT, USA). Since the ATPase activity of ABC transporters is inhibited by sodium orthovanadate (vanadate), the ABCB1-specific ATPase activity was calculated as the difference of the ATPase activities in the vanadate-treated and untreated sample pairs.

### 4.6. UIC2 Reactivity Assay

The ABCB1-positive NIH 3T3 cells (0.5 × 10^6^ mL^−1^ in gl-PBS) were pre-incubated with QUR, C3S, QUR + C3S added at different concentrations or a known ABCB1 inhibitor CsA (10 µM) for 10 min and then further incubated with 10 µg/mL UIC2-A647 monoclonal antibody at 37 °C. After 30 min of incubation, the samples were washed 2 times with ice-cold gl-PBS and centrifuged for 5 min at 435× *g* at 4 °C. The UIC2-A647 fluorescence intensity of the cells was measured by flow cytometry. To exclude dead cells from the analysis, the samples were stained with propidium iodide (PI).

### 4.7. Flow Cytometry

Flow cytometry analysis was carried out using a Becton Dickinson FACS Calibur flow cytometer (Becton Dickinson, Mountain View, CA, USA). A 488 nm laser was used for the excitation of calcein and daunorubicin and the emitted light was detected using a 502 nm dichroic mirror and a 530/30 nm band-pass filter (for calcein), while the fluorescence of PI and daunorubicin was detected by applying a 585/42 nm band-pass filter. The fluorescence signal of A647 was detected using a 635 nm red diode laser and a 661/15 nm band-pass filter. Cell debris was excluded from the analysis on the basis of FSC and SSC signals. Fluorescence signals of 2 × 10^5^ cells/sample were collected in logarithmic mode, and the obtained cytofluorimetric data were analyzed using the Flowing software (version 2.5.1, Cell Imaging Core, Turku Centre for Biotechnology, Turku, Finland).

### 4.8. Statistical Analysis

SigmaPlot (version 14, SSI San Jose, CA, USA) was used for statistical analysis. For the comparison of two samples from normally distributed populations with equal variances, Student’s *t*-test was performed. Multiple comparisons were performed with analysis of variance (ANOVA) applying the Holm–Sidak test for post hoc pair-wise comparison of the data. Differences were considered significant at *p* < 0.05.

### 4.9. Molecular Docking

The cryo-EM structure of human ABCB1 in complex with the inhibitor ZQU (PDB ID: 7A6F, resolution: 3.50 Å) was used. Missing residues (a.a. 1–31, 86–103, 631–693 and 1277–1280) were modeled using Modeller 9.25 [45]. The bound ligand and the water molecules were removed before docking. Then hydrogen atoms were added, and their positions were optimized in Tinker 8 program [46]. The correct protonation states were assigned using PROPKA [47,48]. ZQU, C3S, and QUR structures were generated using Marvin Sketch 5.6.0.0 (http://www.chemaxon.com (accessed on 22 December 2022)). The 3D structures were geometry optimized after assigning Gasteiger charges in UCSF Chimera 1.8 [49] with the combination of steepest descent and conjugate gradient algorithms with 100 steps (step size 0.05 Å), and a conjugate gradient method with 10 steps (step size 0.01 Å).

Molecular docking studies were performed with AutoDock Vina (Version 1.1.2, Scripps Research, La Jolla, CA, USA) [25]. Grid boxes were set up large enough to encompass each pose of ZQU identified in the ZQU-bound ABCB1 cryo-EM structure. For the central drug-binding site (site 1) the grid box dimensions were 14, 12, and 16 Å. For the “access tunnel” (site 2) the grid box dimensions were 9, 15, and 12 Å. The ABCB1 structure was rigid during docking. To avoid any built-in bias related to the initial conformation of ligands upon docking, we opted for the default random position/conformation in AutoDock Vina. In order to validate the docking protocol, two ZQU molecules were docked sequentially at the two binding sites (Figures S1 and S2 and Supplementary Materials).

C3S and QUR were docked at each site using the same protocol. One of these two molecules was docked at site 1 and docking scores were evaluated. The ligand poses were ranked based on their docking score. In the next step, keeping the best pose of this ligand at site 1, the other ligand (C3S or QUR) was docked at site 2 (Figure S3). In order to investigate the entire space of the binding cavity the exhaustiveness was set to 100.

### 4.10. Molecular Dynamics

Three ligand–ABCB1 complexes (2×C3S–ABCB1 complex, C3S-QUR–ABCB1 complex and 2×QUR–ABCB1 complex) were embedded in a 1-phosphatidyl-2-oleoyl-sn-glycero-3-phos-phocholine (POPC) lipid bilayer built with CHARMM-GUI [50,51]. Ligand topologies were generated with CHARMM General Force Field (CGenFF) using the ligand coordinates [52,53]. ABCB1 was placed in the lipid bilayer based on the Orientation of Proteins in Membrane (OPM) database [54]. Thereafter, the system was solvated in such a way that the solvation extended approximately 10 Å at both sides of the protein and neutralized with 150 mM KCl. The final system comprised 199,824 atoms with dimensions of 105 × 105 × 191 Å. GROMACS 2020.4 was used to perform MD simulations using the GPU cluster Komondor (Governmental Information-Technology Development Agency, Budapest, Hungary). The system was initially minimized with a steepest descent integrator until the threshold (Fmax < 10 kJ/mol) was reached. The system was later subjected to 6 equilibration steps, where the first 3 steps of equilibration were performed at 1 fs and the remaining 3 steps were performed at 2 fs integration time steps. The first two NVT equilibration steps were performed using a Berendsen thermostat [55], while later NPT steps used a Berendsen thermostat and a Berendsen barostat for semi-isotropic pressure coupling at 1 bar [56]. The equilibration was performed in triplicate to derive different velocities in each equilibration. The temperature was maintained at 310 K. The ABCB1 protein, the membrane and the TIP3P solvent molecules [57] were separately coupled. Long-range electrostatic interactions were calculated with particle mesh Ewald method [58] with a cut-off of 1.2 nm, while the van der Waals interactions were calculated with a cut-off of 1.2 nm. All bonds were constrained by the LINCS algorithm [59]. The systems with three equilibration states (i.e., different initial velocities) were subjected to a 400 ns production phase of MD simulations using a Nose-Hoover thermostat [60,61] and a Parrinello-Rahman barostat [62].

### 4.11. MD Analysis

Root mean square deviation (RMSD) and root mean square fluctuations (RMSF) of side chain atoms were calculated using GROMACS tools. Residue contact maps, the solvent-accessible surface area (SASA) between ligands and ABCB1, and distance analyses for the minimum distance between ligands and nearby residues were also performed using GROMACS tools (mdmat, sasa, covar, and anaeig) [63]. The number of hydrogen bonds formed between the ligands and ABCB1 residues was analyzed using PyMOL Molecular Graphics System, Version 2.4.1 (Schrodinger, LLC) available at http://www.pymol.org/pymol (accessed on 25 January 2020).

MD frames taken from the concatenated trajectories from the simulation period between 300 and 400 ns with 250 ps steps were subjected to Poisson Boltzmann surface area continuum solvation (MM-PBSA) calculations [64,65] to derive the binding free energy estimates. In MM-PBSA calculations, various energy terms such as van der Waals energy, electrostatic energy, polar solvation energy and SASA energy were evaluated to calculate the binding free energy. Furthermore, the physicochemical traits including dielectric constants of solvent and solute molecules, which were set to 80 and 4, respectively, the radii of positively and negatively charged ions, which were set to 0.95 and 1.81 Å, respectively, and the concentration of these ions, which was set to 0.15 moles, respectively, were taken into account in the MM-PBSA calculations.

## 5. Conclusions

The biochemical experiments as well as in silico studies described herein suggest that C3S and QUR, when co-administered, may bind to the substrate-binding cavity simultaneously, and induce a strong ABCB1-inhibitory effect. When studying the conformational dynamics of different ligand–ABCB1 complexes (2×C3S–ABCB1, C3S-QUR–ABCB1, and 2×QUR–ABCB1), it was demonstrated that the substrate-binding pocket was able to adapt to the size of the different ligand combinations. Comparing the hydrogen bonding partners of the different ligand combinations, it can be concluded that the pattern of hydrogen bonding interactions of a particular ligand at one site is affected by the identity of the ligand occupying the other binding site, further supporting the “substrate induced fit model” formulated a long time ago. Collectively, a combination of functional and in silico studies can improve our understanding regarding the molecular details of ligand–ABCB1 interactions.

## Figures and Tables

**Table 1:** Hydrogen bond occupancy (%) for ABCB1 complex containing C3S at both ligand-binding sites (2×C3S–ABCB1).

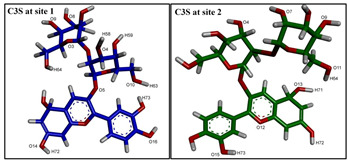			
Hydrogen Bonding Partners in the Central Binding Site (Site 1)	Occupancy (%)	Hydrogen Bonding Partners in the “Access Tunnel” (Site 2)	Occupancy (%)
C3S(H63)—**Gln838** * (OE1) **	19.1	C3S(H63)—**Gln725**(OE1)	35.5
C3S(H63)—Asn721(O)	40.4	C3S(H72)—**Glu875**(OE2)	18.9
C3S(H60)—**Gln725**(OE1)	99.1	C3S(H72)—**Glu875**(OE1)	23.4
Gln838(E21)—C3S(O10)	42.7	**Tyr953**(HH)—C3S(O13)	92.9
Gln838(E21)—C3S(O16)	30.4	**Gln725**(E21)—C3S(O10)	35.4
Gln838(E21)—C3S(O15)	23.3	**Tyr310**(HH)—C3S(O8)	75.5
**Tyr310**(HH)—C3S(O9)	43.5		
**Tyr307**(HH)—C3S(O8)	97.3		
Trp232(HE1)—C3S(O13)	37.2		
Ser228(HG1)—C3S(O14)	22.3		

The image above the table highlights hydrogen bonding atoms of C3S at sites 1 and 2. * The order of hydrogen bonding partners’ labels: donor–acceptor. ** The main amino acid residues forming predominant hydrogen bonds are shown by bold letters.

**Table 2:** Hydrogen bond occupancy (%) for the C3S-QUR–ABCB1 complex.

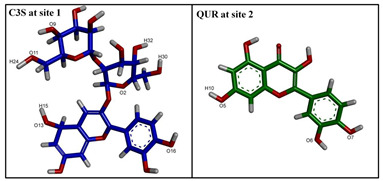			
Hydrogen Bonding Partners in the Central Binding Site (Site 1)	Occupancy (%)	Hydrogen Bonding Partners in the “Access tunnel” (Site 2)	Occupancy (%)
C3S(H32)—**Gln990** * (OE1) **	52.0	Gln347(E21)—QUR(O3)	33.9
C3S(H30)—**Phe983**(O)	88.6	**Try310**(HH)—QUR(O7)	58.8
C3S(H15)—Glu875(OE2)	27.2		
C3S(H15)—Glu875(OE1)	38.6		
**Gln990**(E21)—C3S(O2)	76.3		
**Trp232**(HE1)—C3S(O8)	44.5		
Trp232(HE1)—C3S(O9)	44.6		
Tyr953(HH)—C3S(O14)	38.0		

Image on the top of the table shows hydrogen bonding atoms of C3S at site 1 and QUR at site 2. * The order of hydrogen bonding partners: donor–acceptor. ** The main amino acid residues forming predominant hydrogen bonds are shown by bold letters.

**Table 3:** Hydrogen bond occupancy (%) for the 2×QUR–ABCB1 complex.

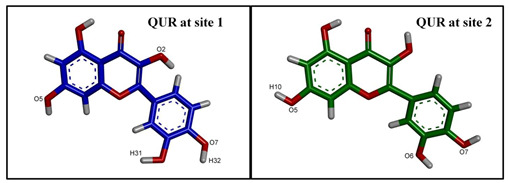			
Hydrogen Bonding Partners in the Central Binding Site (Site 1)	Occupancy (%)	Hydrogen Bonding Partners in the “Access Tunnel” (Site 2)	Occupancy (%)
Tyr953(HH)—QUR *(O6) **	17.7	**Tyr310**(HH)—QUR(O7)	97.5
**Gln725**(E21)—QUR(O2)	32.1	**Ser228**(HG1)—QUR(O5)	93.4
**Gln725**(E21)—QUR(O4)	40.9		

Image on top of the table shows hydrogen bonding atoms of QUR at site 1 and at site 2. * The order of hydrogen bonding partners: donor–acceptor. ** The main amino acid residues forming predominant hydrogen bonds are shown by bold letters.

**Table 4:** Results of MM-PBSA calculations on trajectories extracted from 300 ns to 400 ns MD time intervals.

System	Van Der Waals Energy (kJ∙mol^−1^)	Electrostatic Energy (kJ∙mol^−1^)	Polar Solvation Energy (kJ∙mol^−1^)	SASA Energy (kJ∙mol^−1^)	Binding Free Energy (kJ∙mol^−1^)
**2×C3S–ABCB1 complex**					
site 1	−133.84 (7.85)	−10.00 (2.40)	62.33 (4.04)	−13.89 (0.82)	−95.41 (7.09)
site 2	−133.04 (9.05)	−13.28 (2.64)	73.21 (4.99)	−14.29 (0.70)	−87.37 (8.31)
total	−266.87 (12.72)	−23.30 (3.56)	149.41 (7.33)	−26.29 (1.16)	−166.99 (12.53)
**C3S-QUR–ABCB1 complex**					
site 1 (C3S)	−231.98 (14.12)	−35.86 (6.29)	171.81 (7.89)	−26.61 (0.98)	−122.66 (12.74)
site 2 (QUR)	−219.58 (15.04)	−34.88 (8.21)	181.38 (13.72)	−25.52 (1.00)	−98.60 (1.70)
total	−451.55 (21.97)	−70.75 (8.99)	351.84 (17.06)	−48.40 (1.48)	−218.96 (24.39)
**2×QUR–ABCB1 complex**					
site 1	−201.51 (15.56)	−39.57 (11.11)	193.47 (19.26)	−26.07 (1.16)	−73.70 (18.64)
site 2	−119.67 (8.89)	−11.01 (2.98)	60.21 (6.35)	−13.79 (0.82)	−84.17 (8.17)
total	−321.26 (19.26)	−50.60 (11.35)	263.44 (19.46)	−36.61 (1.70)	−144.93 (21.17)

(SD values are given in parenthesis).

## Data Availability

Data obtained or analyzed during the current study are available from the corresponding author upon reasonable request.

## Supplementary Materials

The following supporting information can be downloaded at: https://www.mdpi.com/article/10.3390/ijms241411341/s1.
