# Supplementary material for: Synergistic Inhibitory Effect of Quercetin and Cyanidin-3O-Sophoroside on ABCB1
Source: Int J Mol Sci. 2023 Jul 12;24(14):11341. doi: 10.3390/ijms241411341 (PMC10379049; doi:10.3390/ijms241411341)
Supplement: Supplementary file 1 [file ijms-24-11341-s001.zip › ijms-2234977-supplementary.pdf]

Supplementary information

1. Validation of the applied docking protocol

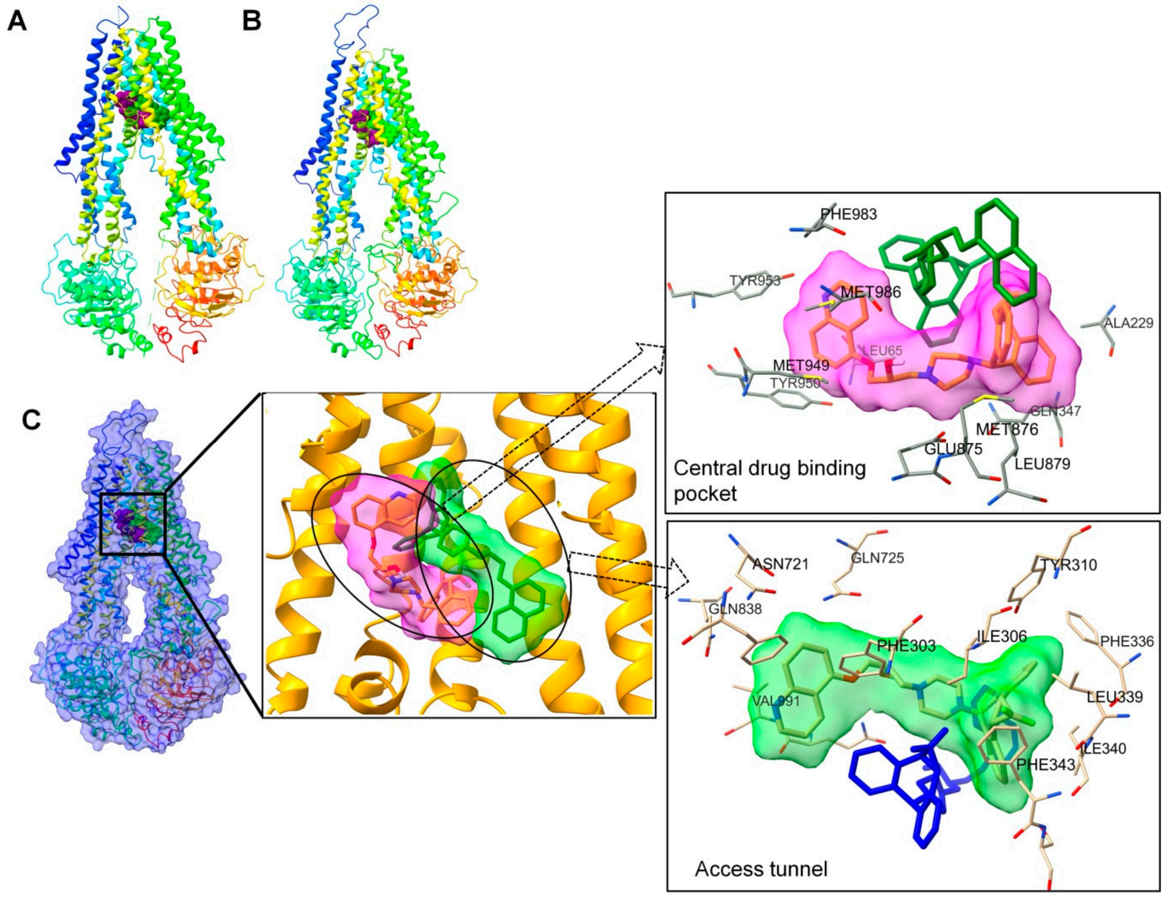

Figure S1. Composition of central drug binding cavity and extended phenylalanine rich "access tunnel" with bound zosuquidar molecules. (**A**) Zosuquidar bound at two sites in the original cryo-EM structure (PDB: 7A6F); (**B**) Structure of ABCB1 with two bound zosuquidar molecules after filling missing residues in loop region; (**C**) Close up view of zosuquidar bound at central drug binding site (site 1) and phenylalanine rich "access tunnel" (site 2).

The 2D structure of zosuquidar (ZQU) was drawn and converted to 3D conformation in Marvin Sketch. The 3D structure was further energy minimized with combination of steepest descent and conjugate gradient criteria in UCSF Chimera. Docking of this optimized structure of ZQU at site 1 and site 2 almost completely reproduced the conformation of the cryo-EM structure of ZQU. The residues interacting with ZQU at site 1 are Ala229, Glu875, Gln347, Leu879, Leu65, Tyr950, Tyr953, Met986, Phe983, Met949 and Met876, while at site 2 the interacting residues are Leu339, Phe336, Gln725, Val991, Gln838, Gln990, Ala987, Phe343, Tyr310, Phe303, Phe770,

Ile299, Phe994, Leu65 and Ser344, as shown in Figure S1. The docking scores of ZQU were 12 kJ/mol and 12.6 kJ/mol for site 1 and site 2, respectively. The root mean square deviations (RMSDs) of docked and cryo-EM poses of ZQU were evaluated to benchmark our docking protocol. In the case of the docked conformer of ZQU at site 1, the RMSD was **0.8639** Å and for the docked conformer of ZQU at site 2, the RMSD was **0.5885** Å. The docked and cryo-EM poses of ZQU are shown in Figure S2. The RMSD values are well below 2 Å indicating that the docking protocol including the setup of grid box dimensions and grid centre are appropriate for further docking simulations. In addition, the docked ZQU molecules show the same kind of interactions with the substrate binding pocket of ABCB1 as it was found in the cryo-EM structure of ZQU-ABCB1 complex further validating our docking strategy.

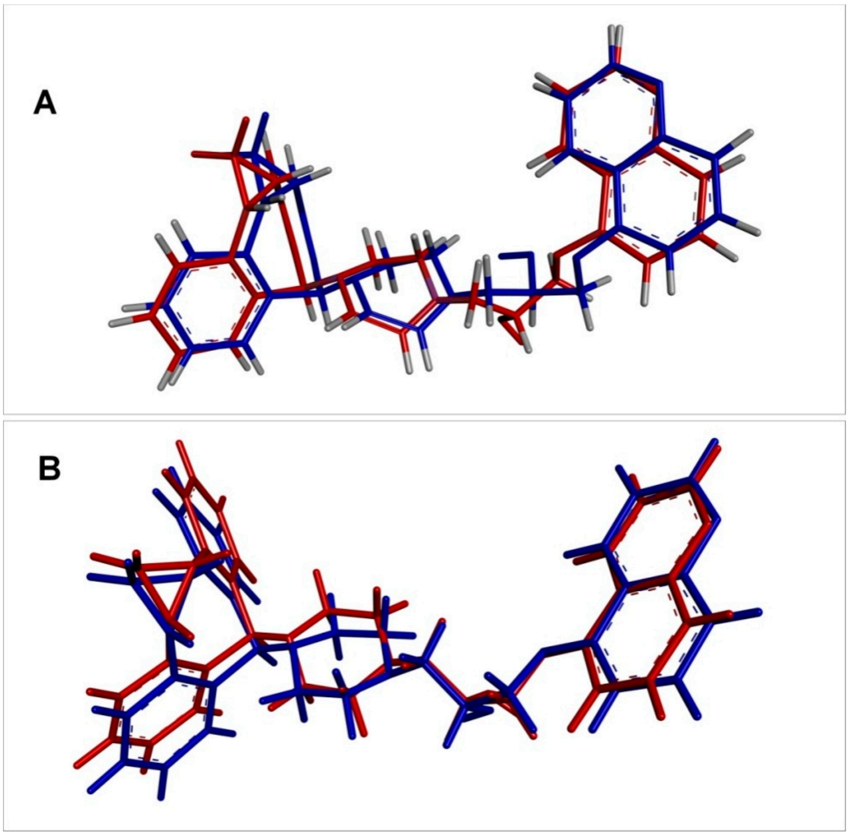

Figure S2: Docked (blue) and cryo-EM (red) poses of Zosuquidar at site 1 (A) and at site 2 (B) in the drug binding cavity of ABCB1.

2. Docking of C3S and QUR

Based on the cryo-EM structure of human ABCB1 in complex with two molecules of the inhibitor ZQU (PDB ID: 7A6F, resolution: 3.50 Å) two ligand binding sites were identified: the central drug binding site (site 1) and the "access tunnel" (site 2). In the first step of docking experiments QUR or C3S was docked at only one site keeping the other site empty (Figure S3). For instance, C3S was docked at the central drug binding site (site 1), which showed a docking score of -7.4 Kcal/mol. Similarly, keeping site 1 empty C3S was docked at the phenylalanine-rich "access tunnel" (site 2), which showed a docking score of -7.7 Kcal/mol. Similarly, when QUR was docked with this approach at site 1 and then at site 2, the docking score was -6.6 Kcal/mol at each site. After this initial guess of possible best docked poses of C3S and QUR at each site, we kept the docked poses of C3S or QUR at either site 1 or site 2 and docked C3S or QUR at the other empty site. For instance, we kept the previously docked pose of C3S (-7.4 Kcal/mol) at site 1 and docked QUR at site 2, which showed a docking score of -8.0 Kcal/mol (See Figure S3). With all possible permutations and combinations, the best ligand pairs and poses were identified. We found that when we kept QUR docked pose with docking score -6.6 Kcal/mol at site 2, the docking of C3S at empty site 1 gave the best and possibly more favourable docked pose of C3S with docking score of -8.4 Kcal/mol. Later, we kept this pose of C3S at site 1 and re-docked QUR at site 2, which gave the docking score of -9.3 Kcal/mol. Thus, we have chosen this ligand-ABCB1 complex (referred as C3S-QUR-ABCB1 complex) for further MD simulations. With similar approach we have selected a ligand-ABCB1 complex with two QUR molecules (2×QUR-ABCB1 complex) with docking score of -7.4 Kcal/mol at site 1 and -9.3 Kcal/mol at site 2 for MD simulations. Interestingly, the ABCB1 complex with two C3S molecules (2×C3S-ABCB1 complex) was found the least favourable compared to the above two ligand-ABCB1 complexes as the docking scores were -5.6 Kcal/mol and -8.0 Kcal/mol at site 1 and site 2, respectively.

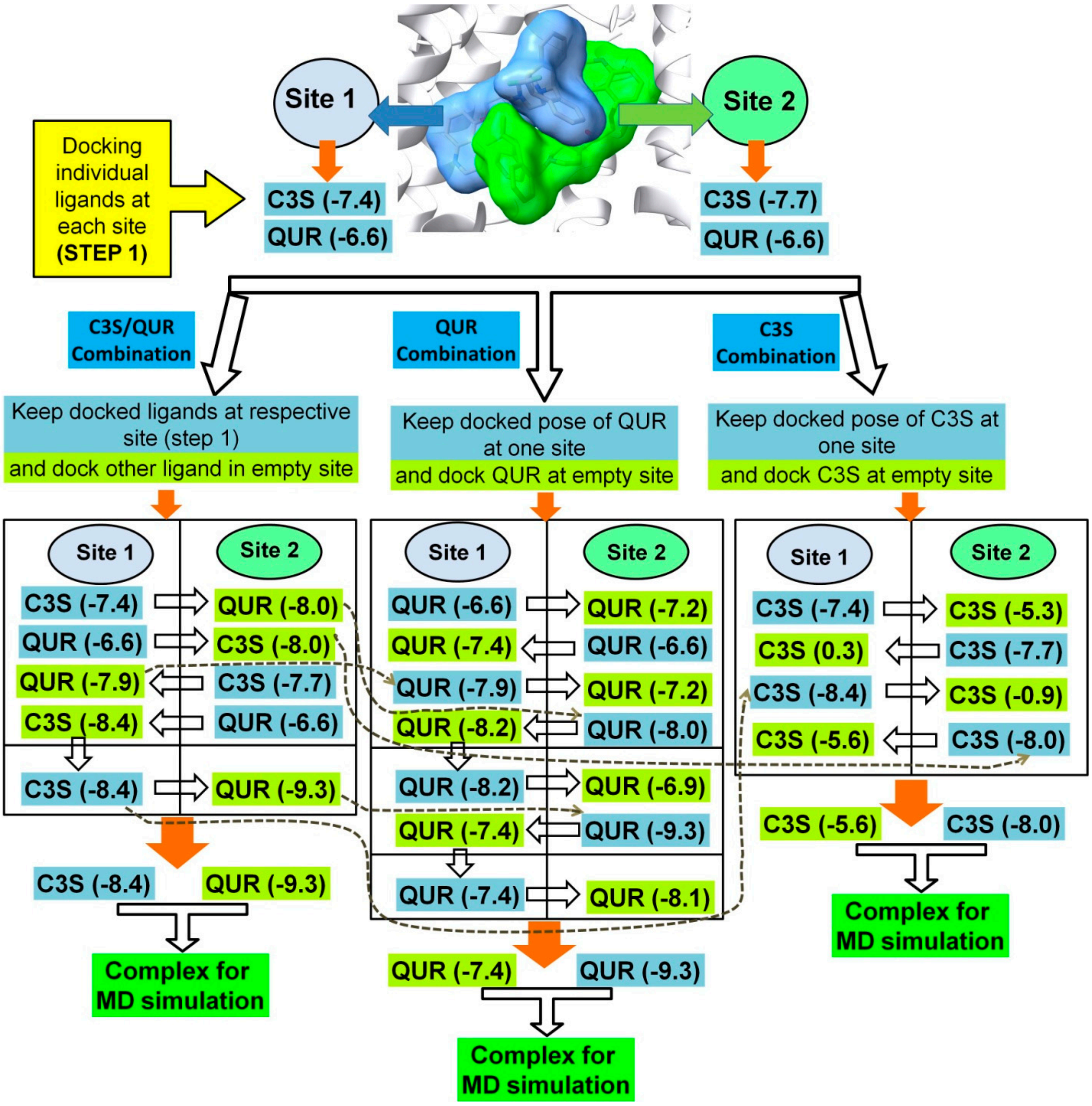

Figure S3. **Docking strategy followed for docking C3S and QUR at site 1 and site 2.** The text highlighted by cyan shows the name and docking score (in parenthesis) of the previously docked ligand at the respective site, while the text highlighted in yellow shows the docking score of the ligand in the respective site in the presence of docked ligand in the other site.

3. Detailed analysis of hydrogen bonding with ligands

2×C3S-ABCB1 complex

In ABCB1 docked with two molecules of C3S, the docked C3S molecule at site 1 showed hydrogen bond interactions with residues Tyr307, Gln990, and Gln725, while C3S at site 2 formed a hydrogen bond interaction with Gln347 and Trp232 (Figure 4A and Figure S4A). After initial energy minimization and six steps of equilibration, in the equilibrated trajectory from run 2 the hydrogen bond interactions with Tyr307, Gln725 and Gln990 present in the docked pose, were broken at site 1 and new hydrogen bond interactions were formed with Ser344 and Glu875. At site 2, the hydrogen bond interactions with Gln347 and Trp232 were also broken and hydrogen bonds with Gln990, Tyr307 and Tyr310 were formed (Figure S4B).

During the production phase of MD simulations, the trajectory extracted at 300 ns showed that the hydrogen bond interactions of C3S at site 1 with Tyr307 and Gln725 were re-established and additional hydrogen bonds with Tyr310, Asn721, and Gln838 were formed (Figure S4C). Interestingly, at site 2, all the hydrogen bonds observed in docked pose and in equilibrated trajectory were broken and a new hydrogen bond was formed with Tyr953 suggesting the onset of large conformational changes in C3S and the surrounding residues (Figure S4C).

The analysis of trajectory extracted at 325 ns revealed that the hydrogen bonds between C3S at site 1 and the residues Tyr307, Asn721 and Gln838 remained stable, while the hydrogen bonds with Try310 and Gln725 were broken (Figure S5A). At site 2, the hydrogen bond with Tyr953 remained stable, while hydrogen bonds with residues Gln725, Gln946, Tyr310 were additionally formed. The trajectory extracted at 350 ns showed that hydrogen bonds with Tyr307, Asn721, Gln838 and Tyr310 remained stable. Furthermore, the C3S molecule at site 1 seemed to extend into the “access tunnel” forming a hydrogen bond with Gln875 (Figure S5B), while the conformation of C3S at site 2 also changed forming hydrogen bonds with residues Ser973 and Glu875 in addition to a stable hydrogen bond with Tyr953. The trajectory extracted at 375 ns showed that the hydrogen bonds between C3S at site 1 and Tyr307, Asn721 and Gln838 remained stable; while a hydrogen bond with Gln725 was re-established as seen in the docked and equilibrated trajectory (Figure S5C). At site 2, the hydrogen bonds with Tyr953 and Glu875 remained intact. The trajectory extracted at the end of MD simulation at 400 ns showed that hydrogen bonds of C3S with Gln725, Tyr307, Gln838 at site 1 and with Tyr953 at site 2 remained stable (Figure S5D). Taken together, in the range of 300 to 400 ns at site 1, hydrogen bonds

between C3S and Tyr307, Gln725, Asn721 and Gln838 are quite stable; while at site 2 only the hydrogen bond with Tyr953 seems to be stable. These MD simulation results corroborate the docking results and highlight that Gln725 and Tyr307 at the site 1 and residues Tyr953 and Glu875 at site 2 are the key residues in hydrogen bond formation.

However, these results are specific to the extracted trajectories at different time intervals of MDS. On the basis of hydrogen bond occupancy evaluation more robust conclusions can be drawn regarding the more prominently existing hydrogen bonds. The network of hydrogen bond pairs with threshold of 15% occupancy is shown in Table 1. As it can be seen at site 1 the residue Gln725, as a hydrogen bond acceptor, forms stable hydrogen bond with C3S with the highest occupancy. The residues Tyr307, Tyr310, Gln838 and Asn721 also form hydrogen bonds with C3S at site 1 throughout the whole simulation period. At site 2, Tyr953 also forms a very stable hydrogen bond with C3S throughout the entire simulation predominantly as a hydrogen bond donor. In addition, the residues Tyr310, Gln725 and Glu875 also form hydrogen bonds with various donor or acceptor atoms of C3S at site 2.

Furthermore, the number of hydrogen bonds formed during the entire MD simulation is also important in characterizing the overall binding interactions between ligands and binding site residues (Figure S6A). The average number of hydrogen bonds formed at the respective site in case of the different ligand combinations is given in Table S1. C3S forms an average of 5 and 3 hydrogen bonds at site 1 and site 2, respectively. Interestingly, the number of hydrogen bonds is slightly fluctuating during the simulation period reaching a value of maximum 7. However, at site 1 the hydrogen bonds are formed consistently throughout the simulation period of 300-400 ns reaching maximum of 10. When both the ligands considered together an average of 6 hydrogen bonds were formed reaching maximum 13 hydrogen bonds in few trajectories.

C3S-QUR-ABCB1 complex

The docked poses of C3S at site 1 showed hydrogen bond interactions with Tyr953, Phe983, Ala987, Gln990 and Glu875, while QUR at site 2 formed hydrogen bond interactions with Gln347, Ser344 and Phe343 (Figure 4B and Figure S7A). After 6 step equilibration the equilibrated trajectory from run 1 showed that C3S formed hydrogen bonds with Gln990, Glu875, Gln4347 and Gln725, while QUR did not show any key hydrogen bond interactions (Figure S7B).

The trajectory of C3S-QUR-ABCB1 complex extracted from concatenated trajectory of triplicate MD simulation at 300 ns showed that C3S at site 1 retained the hydrogen bonds with Gln990, Glu875 and Gln347, while the hydrogen bonds with Tyr953, Phe983 were re-established as seen in the docked pose. In the same trajectory, QUR at site 2 reformed a hydrogen bond with Gln347 and also formed a new hydrogen bond with Tyr310 (Figure S7C). Here, the C3S at site 1 and QUR at site 2 was found to form a hydrogen bond with a common residue Gln347 indicating a close association between the conformations of C3S and QUR. Thus, the pose of C3S seems to extend deeper into the "access tunnel" that is allowed by the smaller molecular volume of the QUR molecule. The trajectory extracted at 325 ns showed that the hydrogen bond interactions between C3S and the Gln990, Glu875 and Phe983 residues of site 1 remained stable from the starting of the equilibration stage. However, a new hydrogen bond is formed between C3S and Trp232 (FigureS8A). In the case of QUR, the hydrogen bond with Tyr310 remained stable. The trajectory extracted at 350 ns showed that the C3S at site 1 retained the hydrogen bonds with residues Gln990, Phe983 and Trp232; while the hydrogen bond with Glu875 broke. At site 2 the hydrogen bond with Tyr310 remained stable (Figure S8B). Notably, at site 2 the hydrogen bond formed between QUR and Tyr310 was broken, while all the hydrogen bonds were preserved at site 1 in trajectory at 375 ns (Figure S8C). The trajectory at end of MD simulation at 400 ns showed that at site 1 the hydrogen bonds between C3S and Phe983, Gln990, Trp232 remained stable and the hydrogen bond with Glu875 was re-established (Figure S8D). However, at site 2 the stable looking hydrogen bond with Tyr310 broke and new hydrogen bonds were formed with Tyr307 and Gln347.

Hydrogen bond occupancy was also studied to describe the number of hydrogen bonds during the entire production phase (Table 2). At site 1, C3S seems to form very stable hydrogen bond of highest occupancy with the residue Phe983, while residues Gln990 Trp232 and Glu875 form hydrogen bonds with different donor or acceptor atoms of C3S. At site 2, QUR forms the most stable hydrogen bond with residue Tyr310 with over 50 % occupancy. Taken together, C3S was observed to be involved in forming an average of 4 hydrogen bonds, while QUR formed around 1 consistent hydrogen bond. When both ligands were considered together an average of 6 hydrogen bonds were formed during the length of 300-400 ns MD simulation period (Table S1 and Figure S6B).

2×QUR-ABCB1 complex

In its docked pose QUR at site 1 formed hydrogen bond interactions with Tyr310, Tyr307 and Gln725, while QUR at site 2 formed hydrogen bonds with residues Ser344 and Gln347 (Figure 4C and Figure S9A). After energy minimization of the system and 6 steps of equilibration run 1 with lowest average RMSD out of the triplicate runs showed that QUR at site 1 formed hydrogen bond interactions with Tyr307, Trp232 and Tyr310, while QUR at site 2 formed no hydrogen bonds (Figure S9B), suggesting that the hydrogen bond with residue Gln725 at site 1 and the hydrogen bonds with residues Ser344 and Gln347 at site 2 were not stable during equilibration. The trajectories extracted at 300, 325, 350, and 400 ns showed that consistent and hence stable hydrogen bonds were formed with Gln725 at site 1 and with Tyr310 at site 2 (Figure S10A-D). Apparently, the conformations of QUR at site 1 and site 2 are quite stable. We have not seen inter-molecular hydrogen bonds between QUR pairs in any trajectories suggesting an independent adaptation of QUR conformations in each binding site (shown in right-hand panel of figure S10A-D). Furthermore, the hydrogen bond occupancy results (Table 3) suggest that at site 1, Gln725 forms a hydrogen bond with highest occupancy. At site 2, the residue Tyr310 forms a hydrogen bond with highest occupancy. Furthermore, the hydrogen bond with Ser228 at site 2 also has high occupancy, although it is not seen in the aforementioned isolated trajectories.

The system with QUR at both of the sites has least number of hydrogen bonds compared to the other two systems. QUR at site 1 and 2 forms only an average of 1 and 2 hydrogen bonds, respectively. When both QUR molecules were considered together an average of 3 hydrogen bonds were formed that is significantly lower compared to the other two systems (Table S1 and Figure S6C).

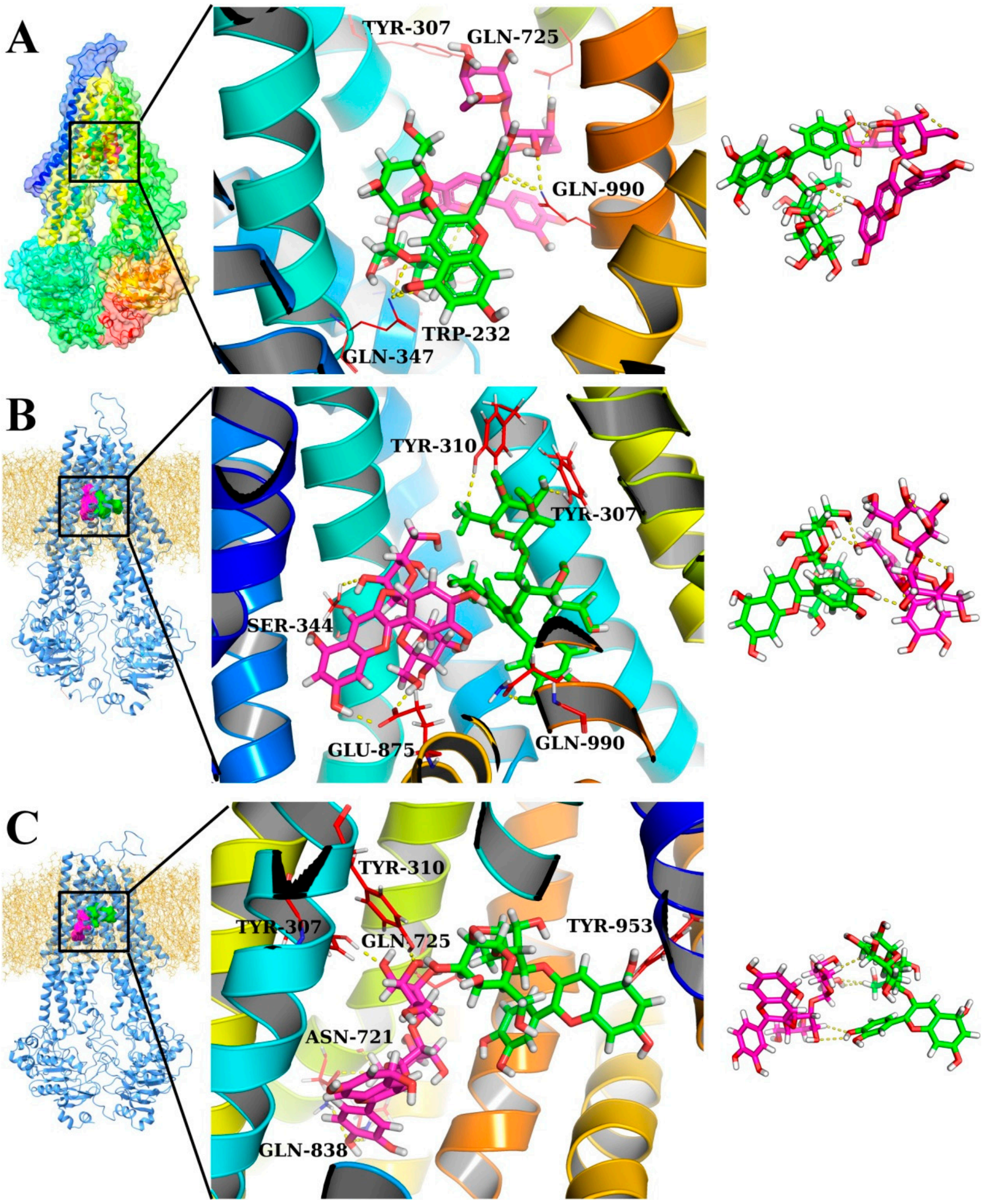

Figure S4. A) Docked complex of ABCB1 with two C3S molecules. B) The trajectory of 6 step equilibration serving as the initial trajectory for production phase MD simulations taken from the run 2 with lowest RMSD. C) The trajectory extracted at 300 ns. The ABCB1 protein is shown either in rainbow or in light blue ribbon and the POPC membrane is shown in light orange line representation. C3S at site 1 is shown in magenta CPK or stick representation, while C3S at site 2 is shown in green CPK or stick representation. The extreme right side of panels show the intra- and inter-ligand interactions

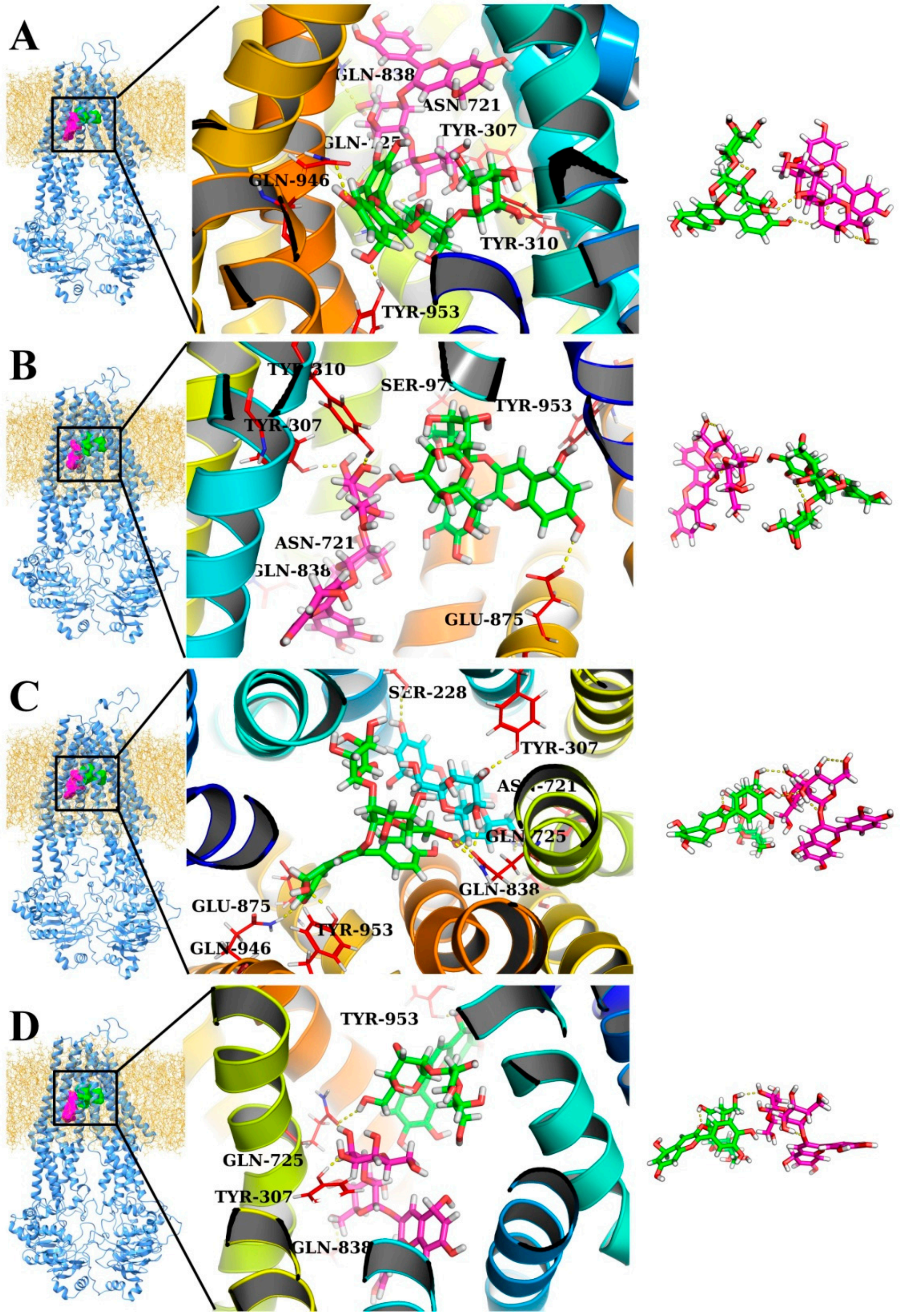

Figure S5. ABCB1 complex with two C3S molecules. The trajectories extracted at **A)** 325 ns, **B)** 350 ns, **C)** 375 and, D) 400 ns MD simulation time periods. The colour schemes are similar to Figure S4.

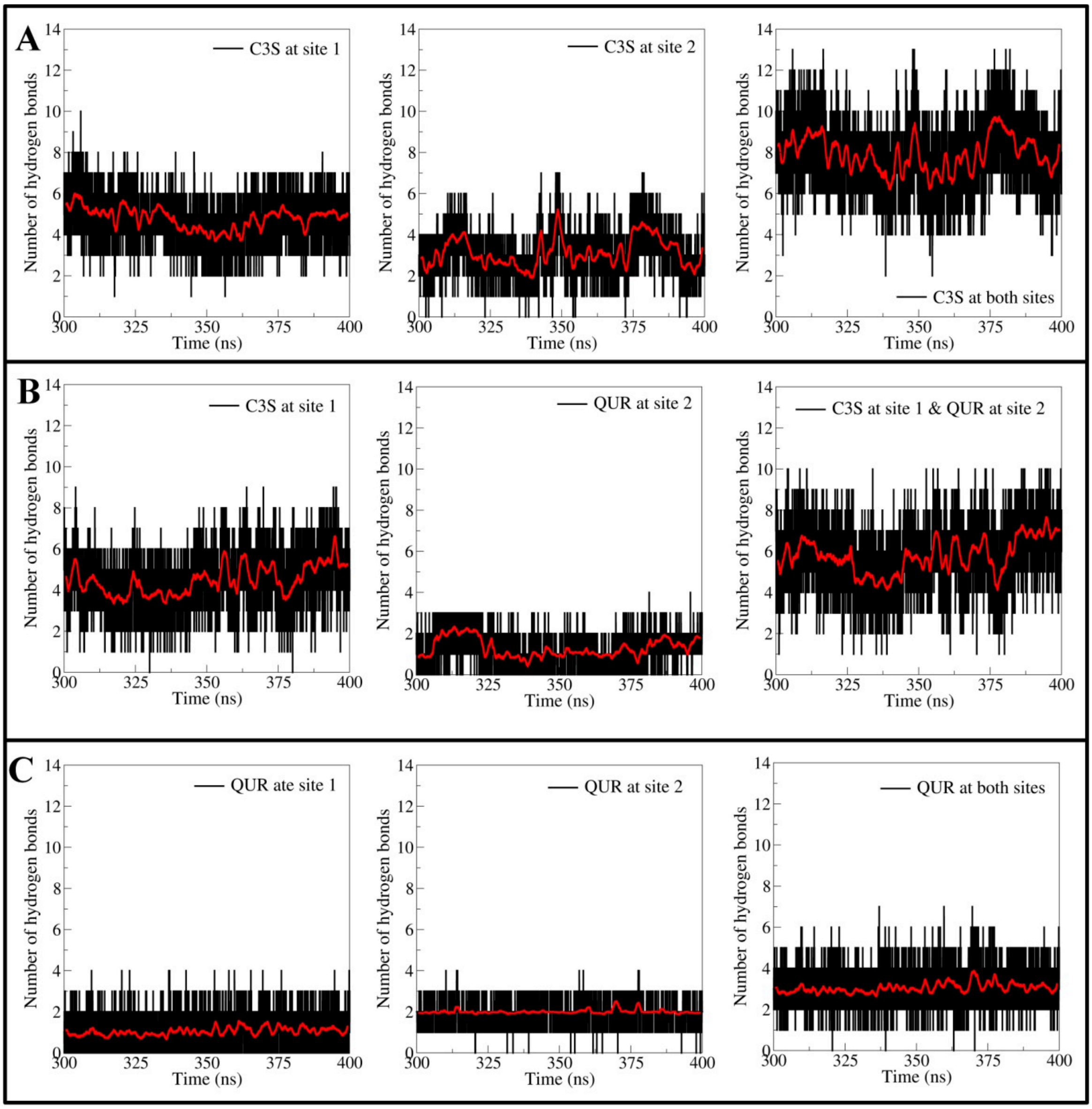

Figure S6. Hydrogen bond analysis. A) 2×C3S-ABCB1complex; B) C3S-QUR-ABCB1 complex and C) 2×QUR-ABCB1 complex

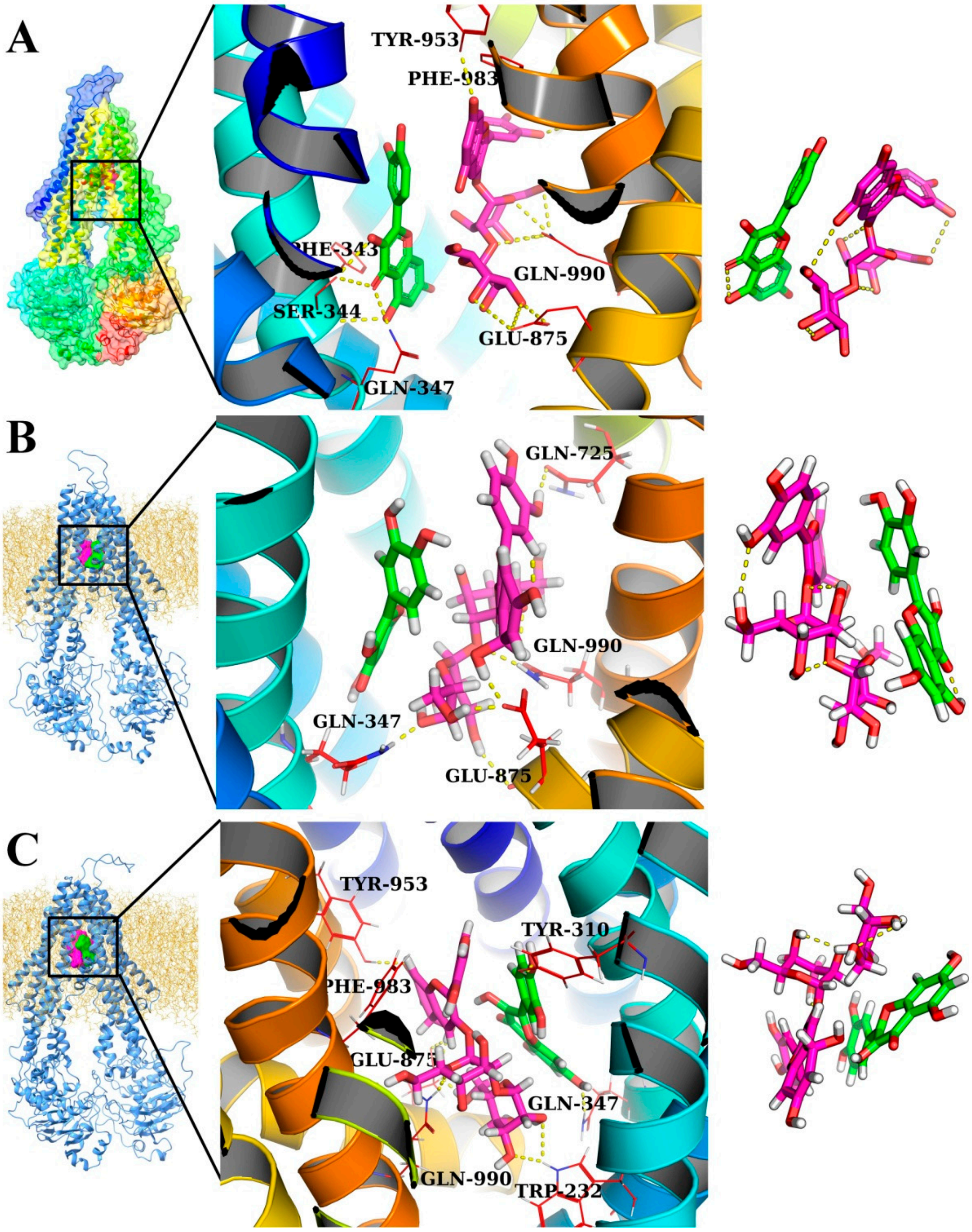

Figure S7. **A) C3S-QUR-ABCB1 complex, where C3S is docked at site 1 and QUR is docked at site 2. B) The trajectory of 6 step equilibration serving as the initial trajectory for production phase MD simulations taken from the run 1 with lowest RMSD. C) The trajectory extracted at 300 ns**. ABCB1 is shown either in rainbow or in light blue ribbon and the POPC membrane is shown in light orange line representation. C3S at site 1 is shown in magenta CPK or stick representation, while QUR at site 2 is shown in green CPK or stick representation. The extreme right side of panels show the intra- and inter-molecular interactions of ligands.

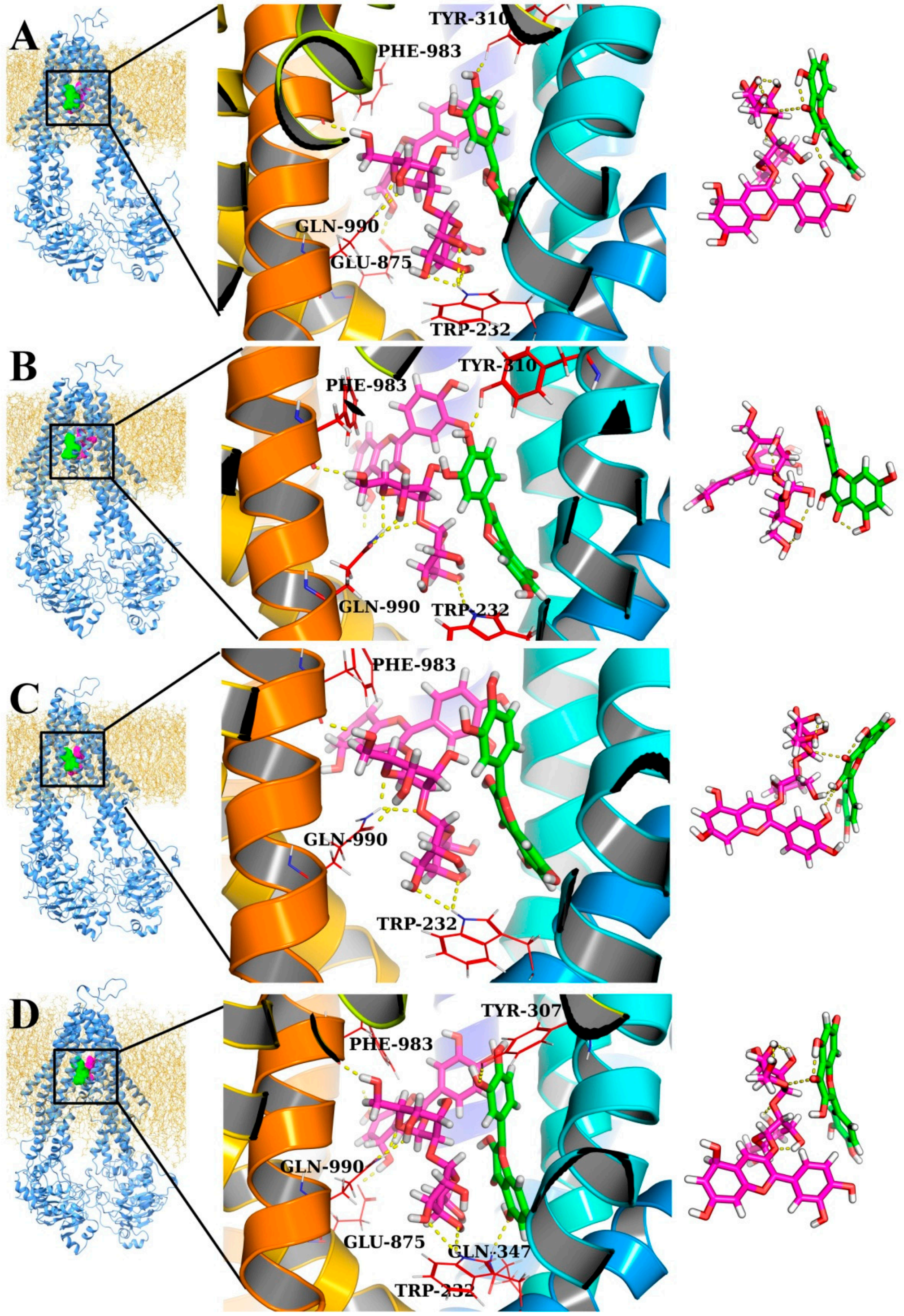

Figure S8. **C3S-QUR-ABCB1 complex**. The trajectories extracted from **A)** 325 ns, **B)** 350 ns, **C)** 375 ns, and D) 400 ns MD simulation time periods. The colour schemes are as stated in Figure S7.

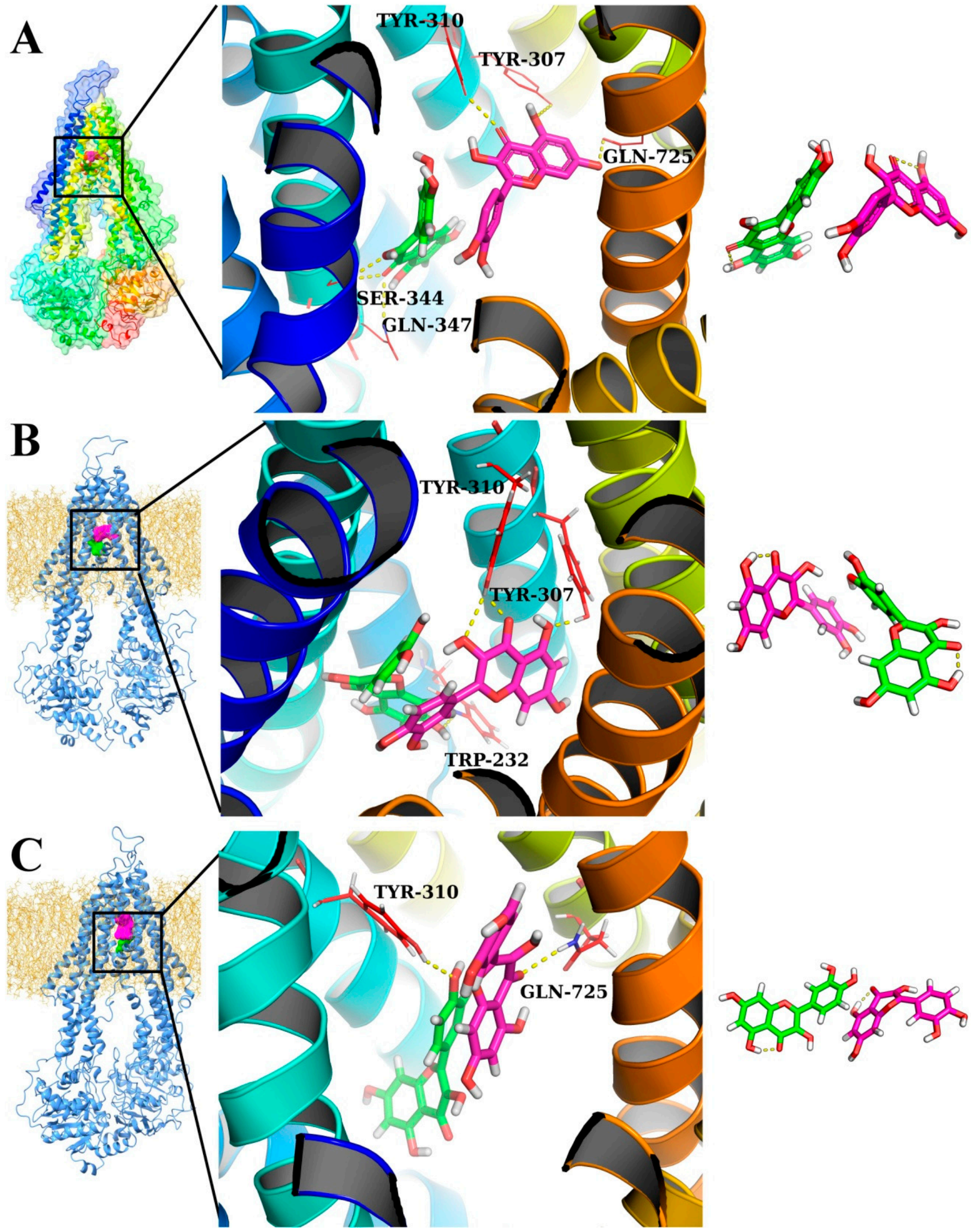

Figure S9. **A) Docked complex of ABCB1witht two QUR molecules. B) The trajectory of 6 step equilibration serving as the initial trajectory for production phase MD simulations taken from the run 1 with lowest RMSD. C) The trajectory extracted at 300 ns.** ABCB1 is shown either in rainbow or in light blue ribbon and the POPC membrane is shown in light orange line representation. QUR at site 1 is shown in magenta CPK or stick representation, while QUR at site 2 is shown in green CPK or stick representation. The extreme right side of panels show the intra- and inter-molecular interactions of ligands.

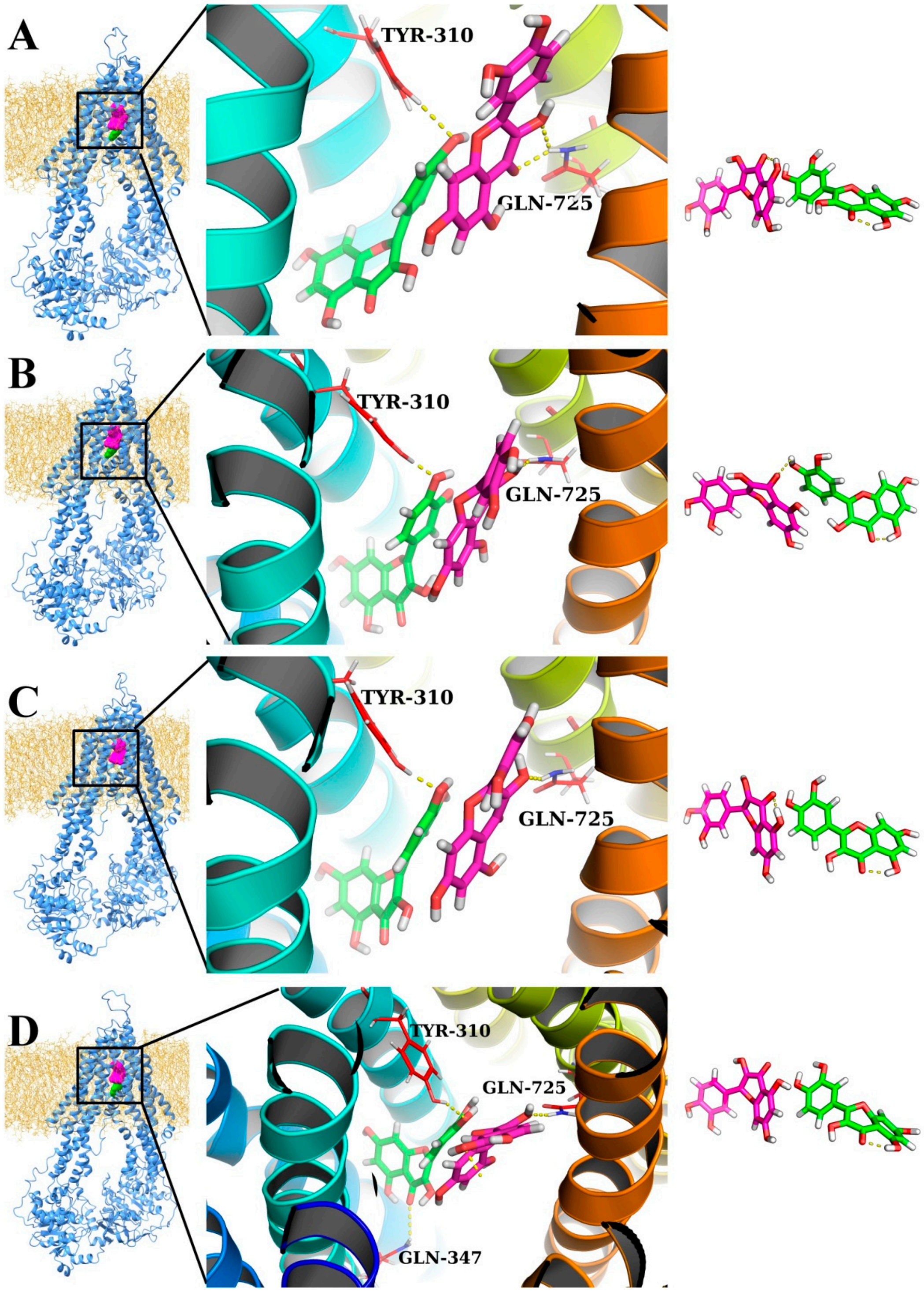

Figure S10. Frames extracted from QUR trajectories at A) 325 ns, B) 350 ns, C) 375 ns, and D) 400 ns MD simulation time period. The colour schemes are as stated in Figure S9.

3.5.2. Studying the stability of the ligand-ABCB1 complexes

To investigate the stability of the entire system both the RMSD of protein C-α atoms and the RMSD of ligand atoms were studied in the triplicate MD simulations of ligand-ABCB1 complexes. RMSD values of C-α atoms of ABCB1 (Figure S11A and Table S1) suggest that the protein structure is the most stable in the 2×C3S-ABCB1 complex with a cumulative average RMSD of 7.17 Å with lowest standard deviation (SD) of 0.83. The lowest SD in the RMSD for the triplicate MD simulation suggests that all runs are almost equivalent. On the other hand, the conformation of the ligand pair is the most stable in the C3S-QUR-ABCB1 complex as the cumulative average RMSD of triplicate run MD simulation for ligand pair at both the sites is 2.33 Å with SD of 0.17 (Figure S11B-D and Table S1).

The analysis of RMSF further assisted in investigating the fluctuations in side chains of amino acid residues. The magnitude of fluctuations in residues of NBD1 and NBD2 are almost similar with an average RMSF of around 5 Å. The RMSF in the transmembrane domains (TMD1 and TMD2) is less pronounced with an average of 2 Å. However, the amino acid residues involved in the formation of the drug binding pocket (residues 300-400 and 900-1000) showed slightly higher fluctuations (Figure S12A).

The nucleotide binding domains (NBD1 and NBD2) and the loop connecting NBD1 with TMD2 (Residue 600-725) exhibited the most significant fluctuations in all ligand-ABCB1 complexes (Figure 6A). Interestingly, the conserved motifs of NBD1 showed the largest fluctuations in the 2×C3S-ABCB1 complex (Figure S12A and S12B), while the complexes with inhibitory ligand combinations (2×QUR or C3S-QUR) exhibited smaller fluctuations in this region. These observations raise the possibility that stabilization of NBD1 may contribute to the ATPase inhibitory effect of QUR and C3S-QUR combination.

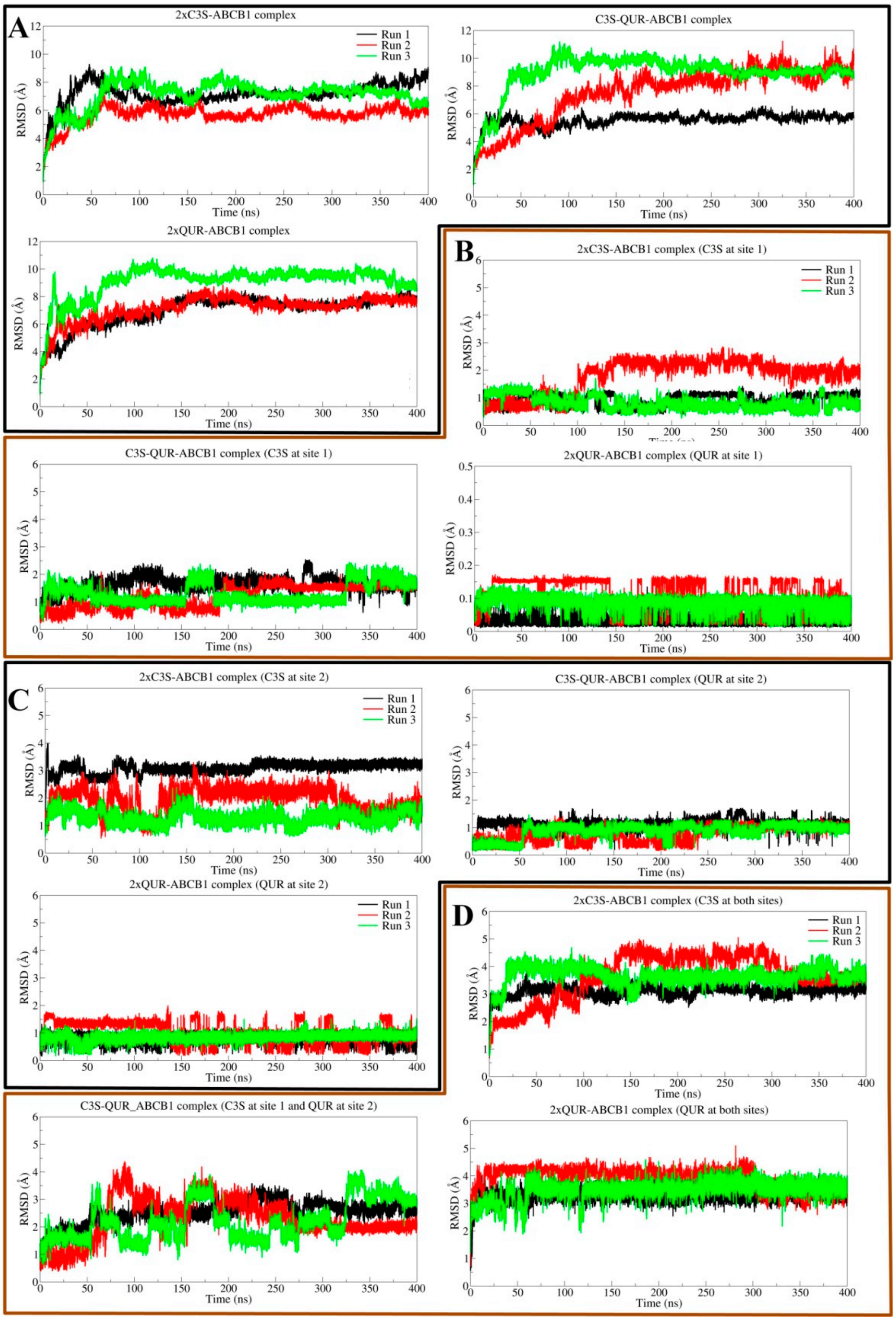

Figure S11. Root mean square deviation analysis. A) RMSD of C-alpha atoms of ABCB1 in systems viz. C3S-combination, C3S-QUR combination and QUR combination, **B**) RMSD of ligand at site 1, **C**) RMSD of ligand at site 2, and **D**) RMSD of ligands at both sites together. Results of RMSD analysis for 400 ns triplicate run MD simulations are shown in each plot.

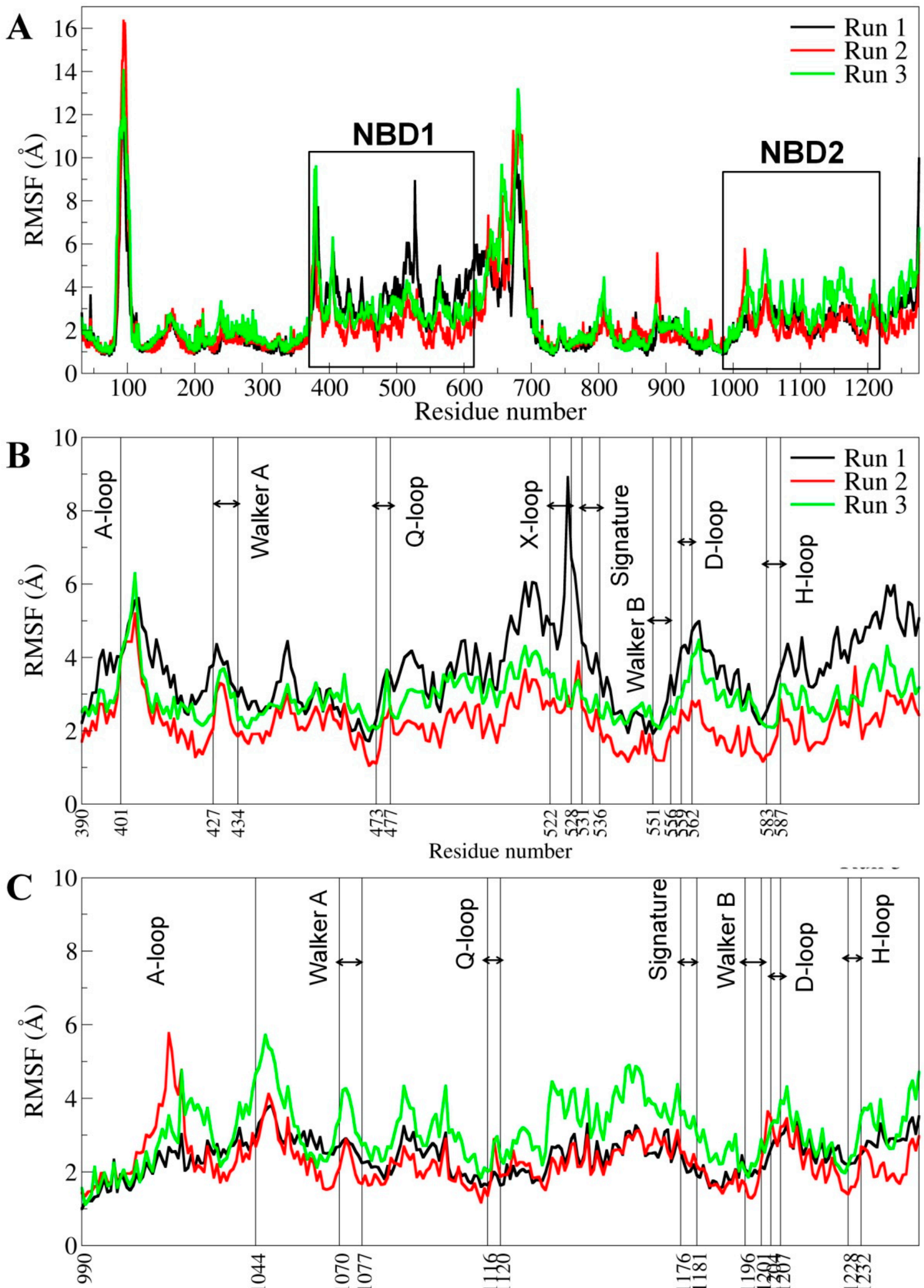

Figure S12. RMSF plots for the 2×C3S-ABCB1complex (A). Zoom in visualization of the NBD1 (B) and NBD2 (C) regions of ABCB1. The characteristic conserved sequence motifs of the NBDs are marked.

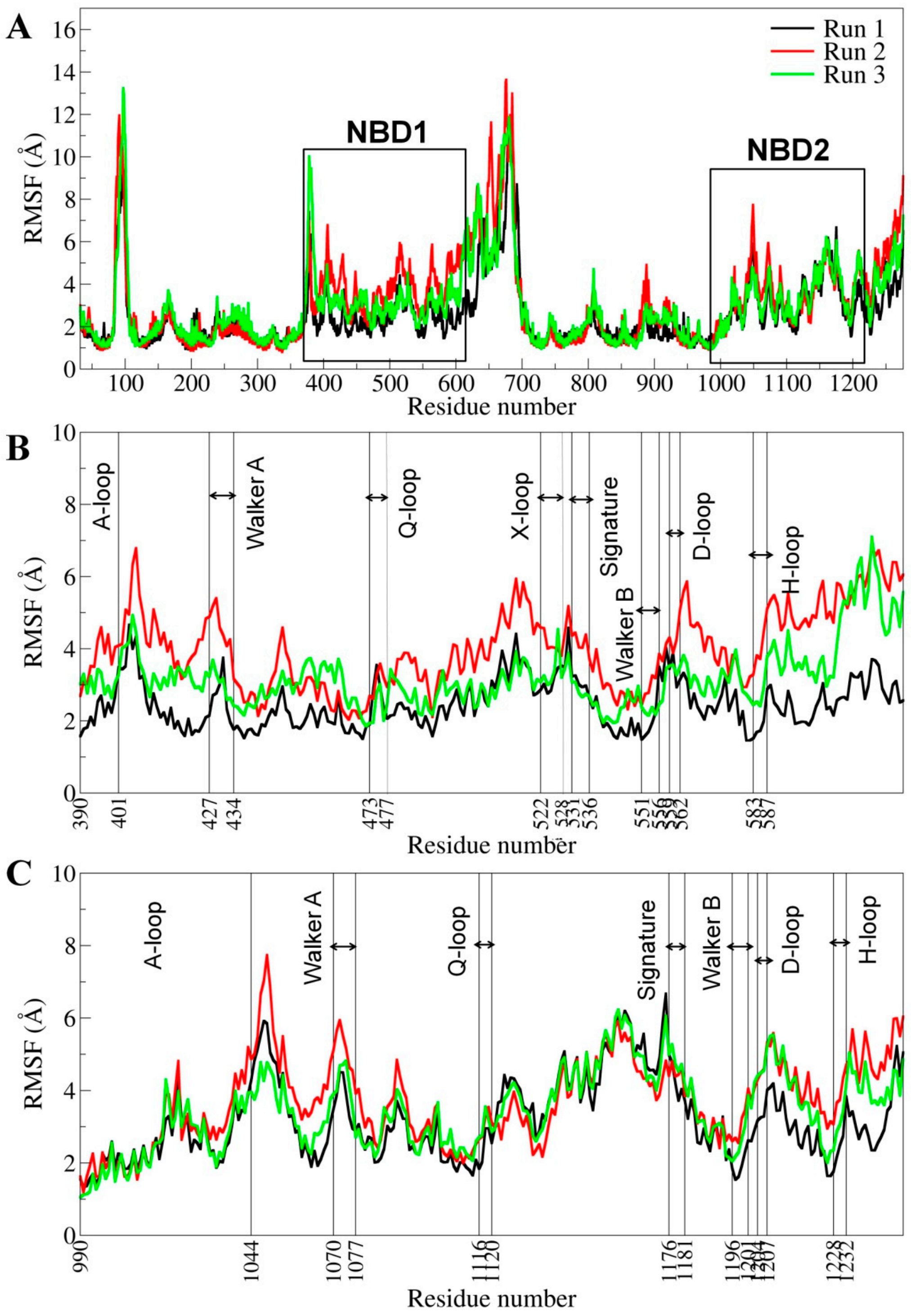

Figure S13. RMSF plots for the C3S-QUR-ABCB1complex (A). Zoom in visualization of the NBD1 (B) and the NBD2 (C) regions of ABCB1. The characteristic conserved sequence motifs of the NBDs are marked.

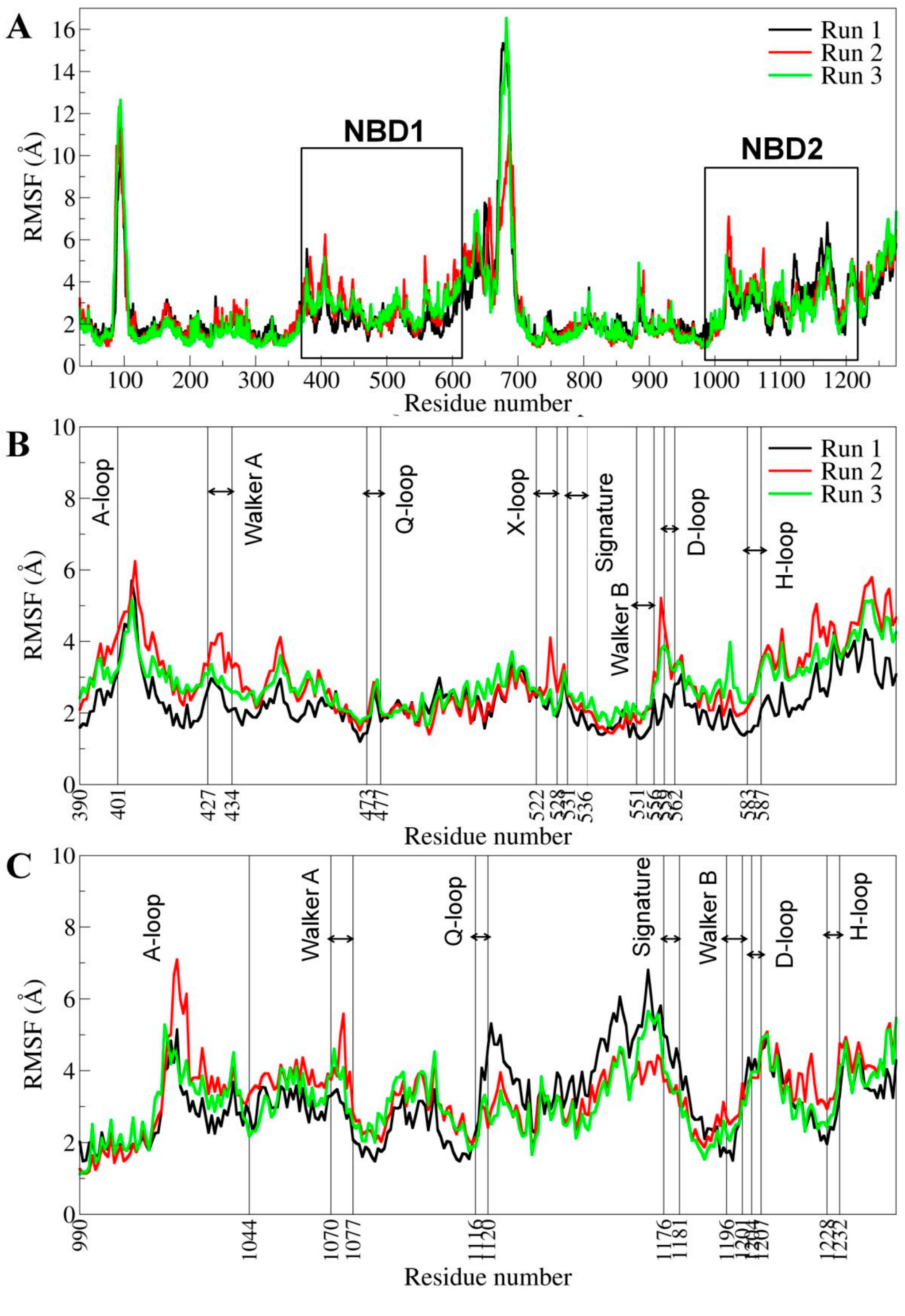

Figure S14. RMSF plots for the 2×QUR-ABCB1complex (A). Zoom in visualization of the NBD1 (B) and NBD2 (C) regions of ABCB1. The characteristic conserved sequence motifs of the NBDs are marked.

.

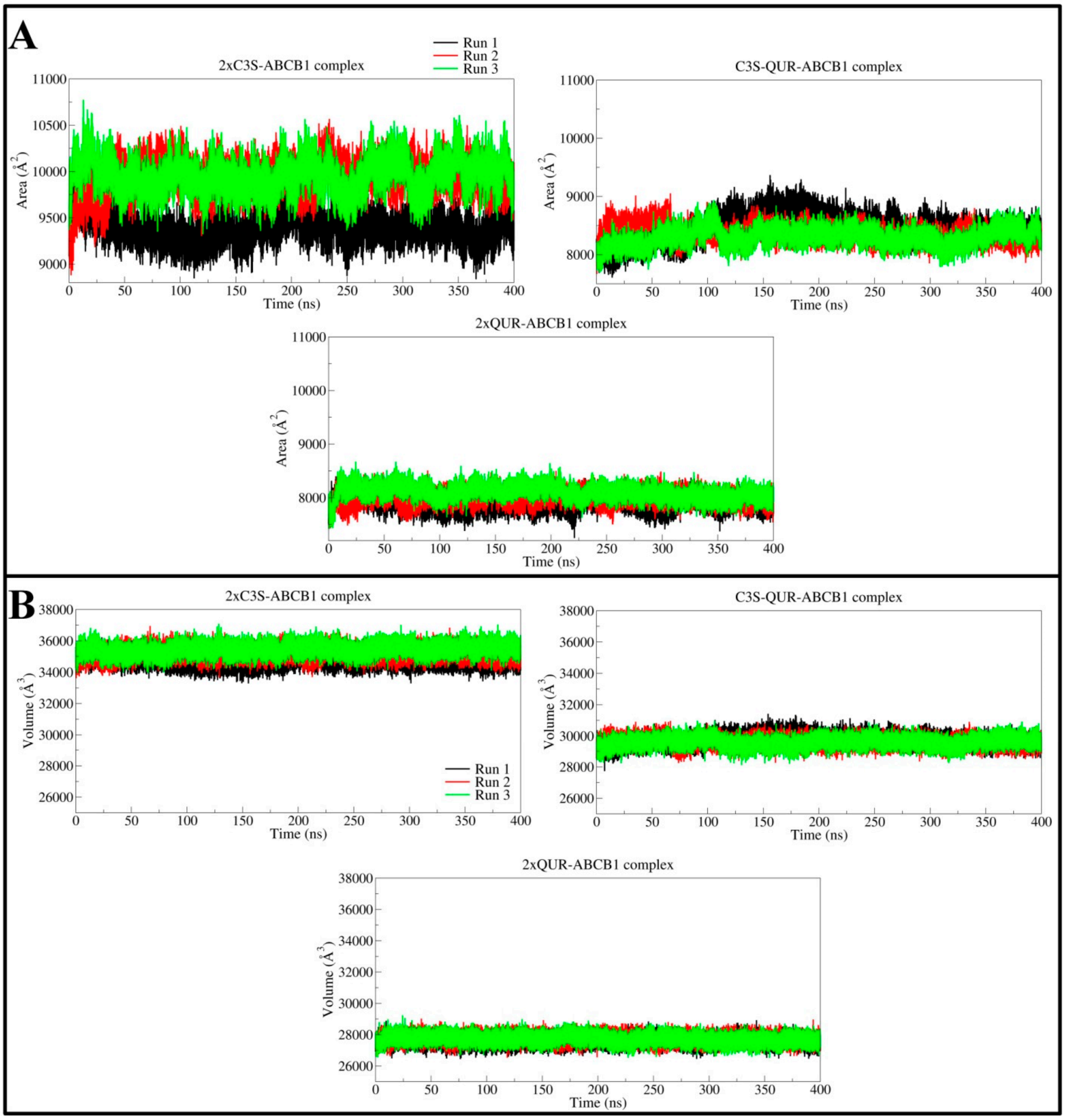

Figure S15. Solvent-accessible surface area plots (A) and volume of ligand binding cavity (B) in the studied ligand-ABCB1 complexes.

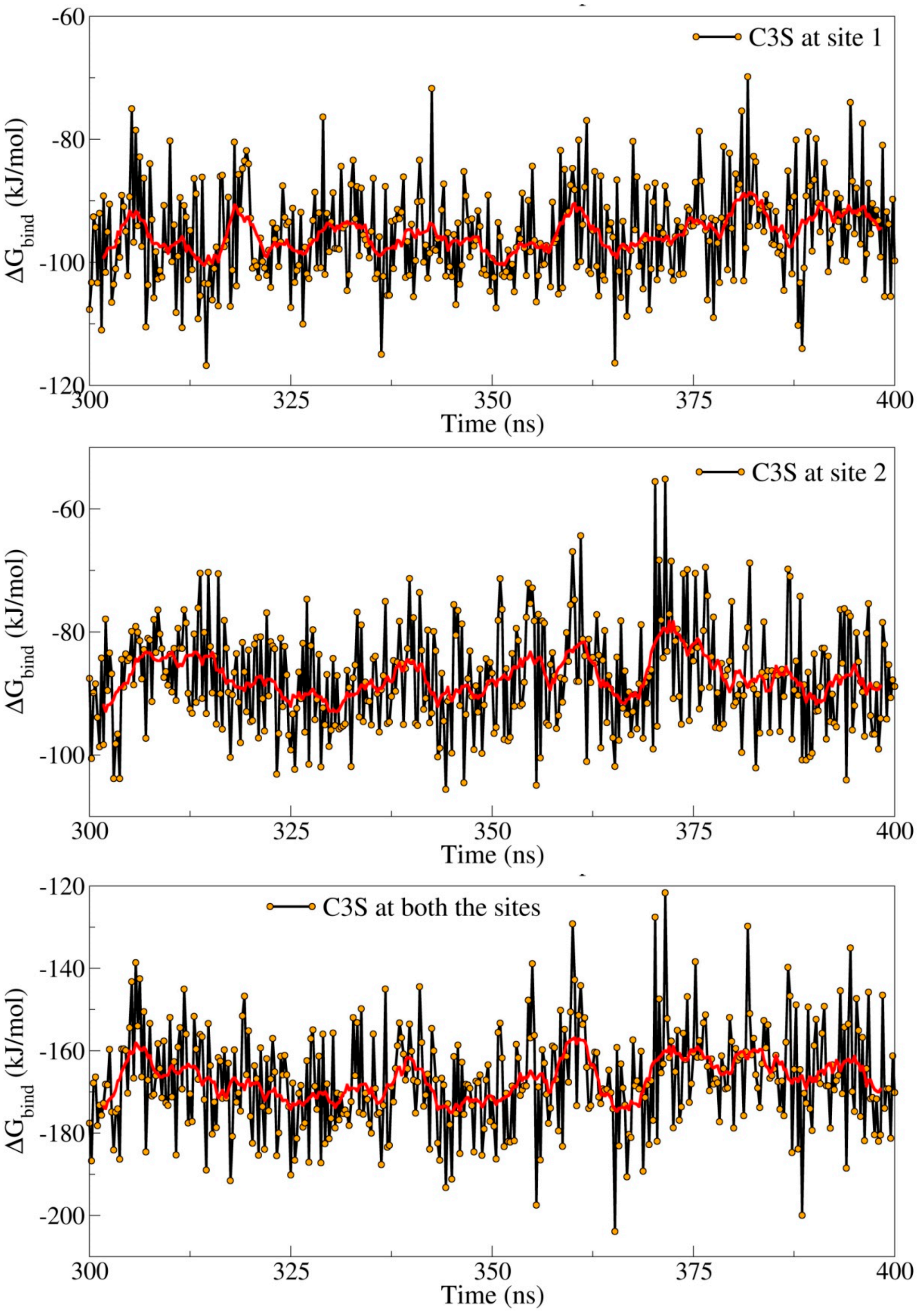

Figure S16. MM-PBSA binding free energies for ABCB1 complex with two C3S molecules (2×C3S-ABCB1 complex). Red lines represent the running averages.

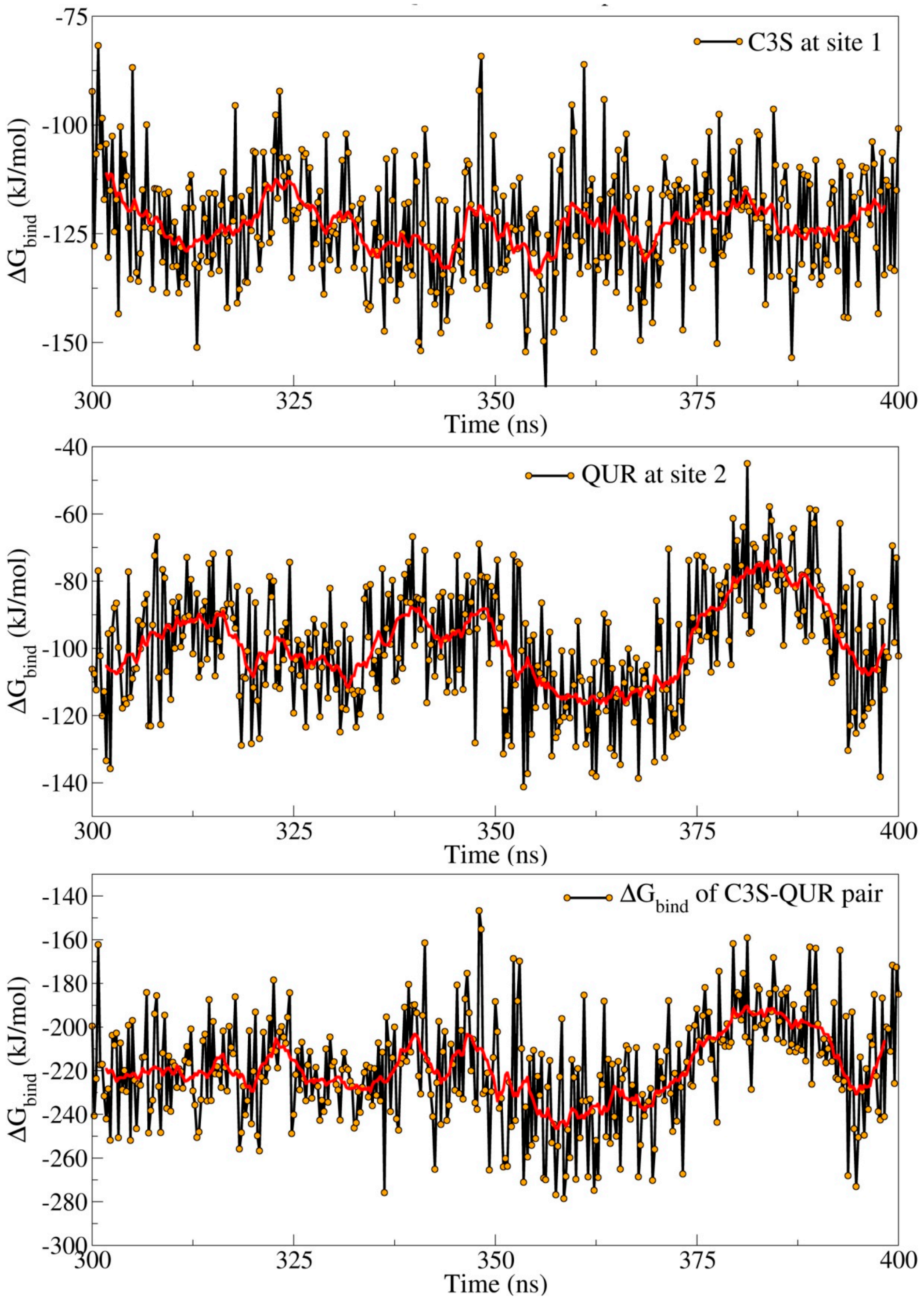

Figure S17. MM-PBSA binding free energies for the C3S-QUR-ABCB1 complex.

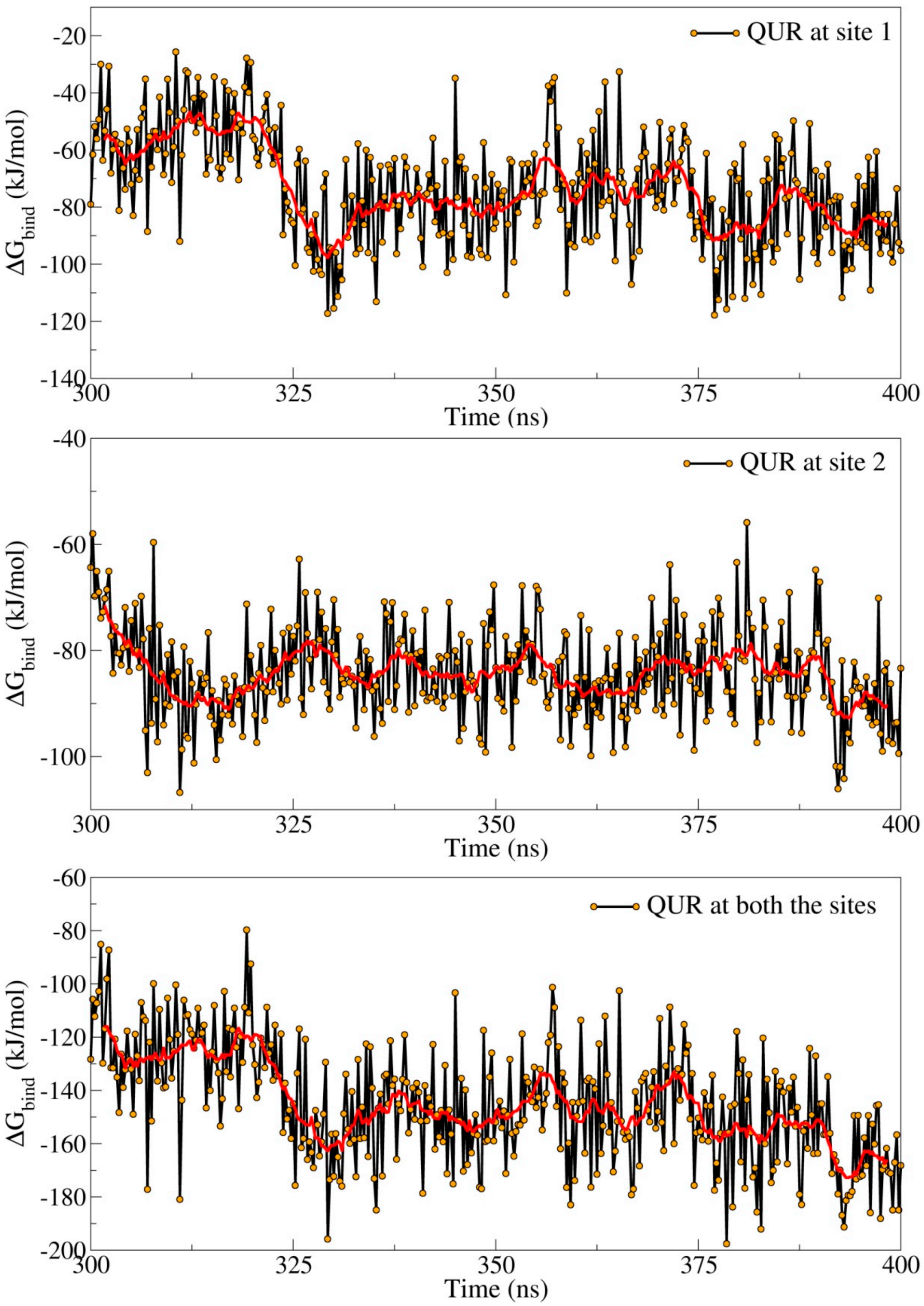

Figure S18. MM-PBSA binding free energies for ABCB1 complex with two QUR molecules.

TableS1. Average, minimum and maximum values of MD simulation parameters

RMSD analysis (Å)	MD simulation Run	2×C3S-ABCB1 complex	C3S-QUR-ABCB1 complex	2×QUR-ABCB1 complex
**Protein C-alpha atoms**				
Average RMSD	Run 1	7.17 (1.12)	5.53 (1.34)	6.96 (1.22)
	Run 2	5.70 (1.19)	7.47 (1.22)	7.12 (1.15)
	Run 3	7.11 (1.23)	8.94 (1.07)	9.06 (1.19)
Cumulative average of triplicate run		7.17 (0.83)	7.31 (1.71)	7.71 (1.16)
**Ligand at site 1**		**C3S**	**C3S**	**QUR**
Average RMSD	Run 1	0.91 (0.52)	1.62 (0.88)	0.31 (0.33)
	Run 2	1.77 (0.59)	1.19 (0.42)	1.12 (0.36)
	Run 3	0.80 (0.67)	1.30 (0.79)	0.85 (0.72)
Cumulative average of triplicate run		1.16 (0.53)	1.37 (0.22)	0.76 (0.41)
**Ligand at site 2**		**C3S**	**QUR**	**QUR**
Average RMSD	Run 1	3.09 (1.18)	1.12 (0.40)	0.78 (0.69)
	Run 2	1.95 (0.91)	0.77 (0.23)	1.06 (0.67)
	Run 3	1.31 (0.74)	0.88 (0.28)	0.84 (0.63)
Cumulative average of triplicate run		2.11 (0.90)	0.92 (0.17)	0.89 (0.14)
**Ligands at both sites**		**C3S-C3S**	**C3S-QUR**	**QUR-QUR**
Average RMSD	Run 1	3.10 (0.97)	2.53 (1.06)	3.26 (0.85)
	Run 2	3.63 (0.97)	2.28 (0.48)	3.90 (0.77)
	Run 3	3.65 (0.96)	2.20 (0.76)	3.47 (2.04)
Cumulative average of triplicate run		3.46 (0.31)	2.33 (0.17)	3.54 (0.32)
**RMSF analysis (Å)**				
Average RMSF	Run 1	2.52 (1.75)	2.57 (1.82)	2.68 (2.22)
	Run 2	2.37 (1.52)	3.16 (1.82)	2.73 (2.32)
	Run 3	2.77 (1.86)	2.94 (1.92)	2.68 (1.77)
Cumulative average of triplicate run		2.55 (0.20)	2.89 (0.29)	2.69 (0.02)
**Solvent accessible surface area (Å^2)**				
Average solvent accessible surface area	Run 1	9385.47 (252.93)	8479.09 (257.26)	7882.48 (134.14)
	Run 2	9924.68 (182.72)	8365.55 (146.61)	7991.20 (126.38)
	Run 3	9951.61 (176.89)	8302.88 (155.08)	8096.06 (135.93)
Cumulative average of triplicate run		9753.92 (319.36)	8382.51 (89.32)	7989.91 (106.79)
**Volume of binding cavity (Å^3)**				
Average volume of binding cavity	Run 1	34722.82 (364.78)	29754.58 (393.35)	27603.34 (299.38)
	Run 2	35211.23 (379.16)	29603.58 (325.05)	27758.39 (296.75)
	Run 3	35456.47 (378.67)	29590.78 (341.58)	27730.47 (303.61)
Cumulative average of triplicate run		35130.18 (373.47)	29649.65 (91.10)	27697.41 (82.64)

**Number of Hydrogen bonds**			
Average number of H-bonds with ligand at site 1	4.79 (1.07)	4.48 (1.26)	1.04 (0.75)
Average number of H-bonds with ligand at site 2	3.12 (1.06)	1.29 (0.76)	2.00 (0.38)
Average number of H-bonds with ligands at both the sites	6.49 (2.74)	5.78 (1.47)	3.04 (0.85)

SD values are given in parenthesis.
